# Interventions for treating posterior cruciate ligament injuries of the knee in adults: A systematic review and meta-analysis protocol

**DOI:** 10.1371/journal.pone.0333015

**Published:** 2025-09-25

**Authors:** Jorge Sayum Filho, Marcel Jun Sugawara Tamaoki, Rogerio Teixeira de Carvalho, Álvaro Nagib Atallah, Fabio Teruo Matsunaga, Flávio Faloppa, João Carlos Belloti

**Affiliations:** 1 Department of Orthopaedics and Traumatology, Universidade Federal de São Paulo, São Paulo, São Paulo, Brazil; 2 Cochrane Brazil, Centro de Estudos de Saúde Baseada em Evidências e Avaliação Tecnológica em Saúde, São Paulo, São Paulo, Brazil; 3 PPG Cirurgia Translacional - Universidade Federal de São Paulo, São Paulo, São Paulo, Brazil; IRCCS Istituto Ortopedico Rizzoli, ITALY

## Abstract

**Introduction:**

There are many different surgical and conservative interventions for treating posterior cruciate ligament (PCL) injuries of the knee in adults. However, in the literature, there is no consensus regarding the best intervention for treating these patients. The objective of this systematic review and meta-analysis is to analyse the effectiveness of interventions (surgical and conservative) for treating PCL injuries of the knee in adults.

**Methods and analysis:**

Starting in November 2025, the authors will accomplish a detailed search using the MEDLINE/PubMed, EMBASE, Cochrane Central Register of Controlled Trials and LILACS databases. Relevant gray literature (academic papers, reference lists theses, technical reports and conference abstracts) will also be included.

Two authors will independently screen and extract the information found from randomized controlled trials in the literature. The bias and quality of the included studies will be evaluated using the Risk of Bias 2 tool provided by the Cochrane Collaboration. Statistical analyses will be performed using Review Manager V.5.4/Review Manager Web software.

**Discussion:**

This systematic review is aimed at providing practical information for Orthopaedic surgeons on the effectiveness of the interventions (surgical and conservative) for treating PCL injuries of the knee in adults.

**PROSPERO registration number:**

CRD42023430493.

## Introduction

The knee is a complex joint formed by the tibia, patella, femur and its ligaments. Ligaments are fibrous connective tissue structures made of collagenous fibres that attach or connect bones to other bones, establishing joints [1–6]. The posterior cruciate ligament (PCL) is the strongest and largest intra-articular ligament of the knee [1,3]. The most important function of the PCL is to serve as the main static stabilizer against posterior translation of the tibia in relation to the femur [1,6–8]. The true incidence of PCL lesions (or injuries) in the world is not known. PCL tears represent 0.64 to 3.3% of all sports-related knee injuries [3,9,10].

The most common mechanism of posterior cruciate ligament lesion is direct trauma in motor vehicle accidents (dashboard injuries – high-energy mechanism). Isolated PCL injuries and incomplete lesions are commonly caused by accidents with low energy, such as hyperextension of the knee. PCL injuries represent 20% of all knee ligament injuries and occur more often in males [3,11].

The diagnosis of posterior cruciate rupture (injury or lesion) is made from the patient’s history, a complete physical examination and imaging exams (X-rays, computed tomography scan and magnetic resonance images) [3,9,12]. PCL injuries may be treated conservatively or surgically. Conservative (nonsurgical) treatment is typically used for less severe lesions, such as partial and isolated PCL injuries and avulsion lesions without displacement [3,13].

Nonsurgical treatment involves immobilization of the leg in nearly full extension for four to six weeks using long‐leg plaster or another type of cast or brace [8]. Patients are generally allowed to partially weight‐bear during this time, and most of them use crutches to ambulate. The possible conservative complications are loss of knee movement, arthrofibrosis, pain, stiffness, a late return to sports activities, thromboembolism, residual instability, etc [3,6,13]. In the literature, there are little existing consensus that isolated PCL injuries of low grade are better treated conservatively. Without evidence, many physicians choose the nonsurgical treatment for elderly and for isolated PCL lesions [6,13].

Surgical intervention generally entails reconstruction or repair of the PCL and other associated lesions (for example, meniscal ruptures and other ligament injuries). It is used for more severe injuries, such as complete nonisolated PCL lesions [3], acute PCL lesions with tibial translation of more than 12 or 13 mm, associated meniscal tears (repairable), bony avulsions, knee dislocations, combined capsuloligamentous injuries, and open fractures [3,13,14]. In the literature, there are little existing consensus that PCL injuries of high grade and injuries with multiple ligament lesions are better treated surgically. Without evidence, many physicians choose the surgical treatment for younger patients and for PCL lesions with associated other injuries (ligaments, meniscal, fractures and others) [3,7].

The reconstruction of the PCL involves many techniques (single band technique, double band technique, isometric point, anatomical point, transtibial tunnel, tibial in lay techniques, high tibial osteotomy for varus, and others) [7,15,16]. It can be performed by open surgery or arthroscopically.

PCL reconstruction can involve the use of many kinds of grafts (e.g., the patellar tendon, hamstring tendons, quadriceps tendon, allograft, and autograft) [3,13,14,17] and many hardware options (interference screws, transverse screws, EndoButton and others). These hardwares can be made of several different materials (metallic, titanium, absorbable, or nonabsorbable materials, plastics and others) [3,14,18]. The possible surgical complications are neurovascular injuries, fractures, infection, residual instability, osteoarthritic progression, osteonecrosis, stiffness, thromboembolism, arthrofibrosis, iatrogenic lesions, etc [3,19,20].

Conservative and surgical interventions have advantages and disadvantages for treating PCL injuries [3,5,19,20]. In the literature, there is no consensus regarding the best intervention for treating PCL lesions [3,13–15]. There is no information whether conservative treatment is better than surgical treatment, there is a lack of evidence in which is the best surgical intervention and in which is the best conservative intervention for treating PCL injuries [3,7].

There are several randomized controlled trials comparing different interventions for treating PCL injuries. However, there is no systematic review of the evidence (with only randomized trials) to inform practice on the best intervention for treating PCL lesions.

Therefore, to assess the effectiveness of interventions for treating PCL injuries in adults, this systematic review is needed.

### Objectives

This review aims to analyse the effectiveness (benefits and harms) of the interventions (surgical and conservative) for treating PCL injuries of the knee in adults [5,21].

## Methods

### Study guidelines and registration

The authors will execute this systematic review and meta-analysis following the Cochrane Handbook for Systematic Reviews of Interventions, and they will report it in accordance with the Preferred Reporting Items for Systematic Reviews and Meta-Analyses (PRISMA) statement [21–26]. This review will start at 2 November2025, and December 2026 is the completion date.

A preestabilished written protocol is registered on the PROSPERO platform (number CRD42023430493) [21].

### Patient and public involvement

This protocol is for a systematic review and meta-analysis; therefore, it will only analyse randomized clinical trials.There is no need for informed consent because there is no patient involvement [21].The target of this systematic review is to ensure that this study is relevant and useful for both patients and healthcare professionals involved in the treatment of posterior cruciate ligament injuries. The authors plan to invite patients with posterior cruciate ligament injuries of the knee, and healthcare professionals with extensive experience in the treatment of posterior cruciate ligament lesions to only provide feedback on the introduction, review findings and conclusions [22].

The consumer involvement (Patient and Public) will be important in ensuring that the review is accessible, clear and accurately reports the most relevant information for both patients and healthcare professionals [22].

### Ethics and dissemination

Ethics approval was not required because the authors will only include published literature. Findings will be disseminated through peer-reviewed publications after data extraction and analysis. The authors plan to complete the searches and analyses by 01 December 2026.

### Eligibility criteria

The authors will only include randomized clinical trials performed in humans, and comparing any interventions for treating PCL injuries of the knee in adults. There will be no restrictions on the language or the year of publication [21].

#### Population.

The authors will include trials involving adults (“≥18 years”, at this age the bone growth lines are closing or are closed what modify the treatment between children and adults) who were diagnosed with posterior cruciate ligament injuries (unilateral or bilateral) of the knee confirmed by magnetic resonance (MR) imaging.

#### Intervention.

Any surgical or non-surgical treatment used for treating PCL injuries that were included in the randomized trials as “intervention”. Randomized clinical trials comparing any methods of operative and conservative (nonsurgical treatment) interventions and comparisons with each other for the treatment of PCL injuries in adults [5]. The interventions above were chosen because they contemplate all interventions to treat posterior cruciate ligament injuries of the knee that may exist in the literature.

#### Comparator.

Any surgical or non-surgical treatment used for treating PCL injuries or no treatment considered in the randomized trials as “control”.

#### Outcomes.

*Primary outcomes:* 1-Validated health-related quality of life scores (36-item Short Form (SF-36); (Ware) or others). 2-Validated patient-rated measures of knee function (the Lysholm Knee Scale, Cincinnati Knee Rating Scale or others). 3- Pain (Visual Analog Scale (VAS) score, Wong-Baker pain scale or others) [27–31].

*Secondary outcomes:* 1- Observer-rated measures of knee function (Roos; Tegner, and others). 2- Knee strength and range of motion. 3- Patient satisfaction with treatment. 4-Infection. 5-Treatment failure. 6-Time of rehabilitation and time to return to previous activities (labour, sports and others). 7-Adverse outcomes: early complications (wound infection or dehiscence, arthrofibrosis, paraesthesia, pain, loss of knee movement, and others); short-term complications (skin problems, stiffness, hardware irritation and loosening, loss of knee movement); and late complications (arthrofibrosis, sensitive or painful wound site, hardware irritation or loosening, loss of knee movement). 8-Radiographic outcomes: widening of the femoral and tibial reconstruction bone tunnels, deformities, loosening of the screws and others [27–31].

If data are available, outcome data for the following time periods will be extracted: short-term follow-up (up to six weeks following treatment), intermediate follow-up (more than six weeks and up to six months after the end of treatment) and long-term follow-up (greater than six months after the end of the intervention) [5,21].

#### Study types.

This systematic review and meta-analysis will only include randomized controlled trials (RCTs). Other studies will be excluded [5,21].

#### Search strategy.

The authors will search the electronic databases until November 2025 for published literature of RCTs to identify eligible studies. These include the PubMed, Cochrane Library, LILACS and EMBASE databases [5,21].

#### Searching other resources.

The authors will search reference lists from all included articles, reviews and textbooks for possible relevant studies, and they will contact experts in the field. They will search for relevant reviews on the database of Health Technology Assessment (HTA) and the Database of Abstracts of Reviews of Effects (DARE), and any errata or retraction from the eligible trials on PubMed, and they will report the date of the search. The authors will also search conference proceedings of the American Academy of Orthopaedic Surgeons and search for gray literature (such as conference abstracts, theses and technical reports) on ProQuest Dissertations and Theses and www.opengrey.eu [32,33].

This search strategy will be modified as required for other electronic databases [19,30,31].

Tables 1–3 electronic search strategy.

**Table 1:** Eletronic search strategies presented to the main databases.

(MEDLINE/PUBMED)
Search Strategy: 1 Posterior Cruciate Ligament/ 2 ((posterior and cruciate and ligament) or PCL).tw. 3 1 or 2 4 exp Surgical Procedures, Operative/ 5 exp Orthopedic Fixation Devices/ 6 Orthopedic Procedures/ 7 (surg* or operat* or reconstruct* or replace* or repair* or graft*).tw. 8 su.fs. 9 or/4–8 10 exp Physical Therapy Modalities/ 11 exp Rehabilitation/ 12 exp Immobilization/ 13 exp Anti-Inflammatory Agents/ 14 Conservative Treatment/ 15 Braces/ or Crutches/ 16 (non-surg* or nonsurg* or non-operat* or nonoperat* or conserv* or rehab* or physiotherapy or physical therapy or exercis* or brace* or cast* or plaster or crutches or immobli* or anti-inflammatory or non-steriod).tw. 17 or/10–16 18 9 or 17 19 3 and 18 20 randomized controlled trial.pt. 21 controlled clinical trial.pt. 22 randomi?Ed.ab. 23 placebo.ab. 24 drug therapy.fs. 25 randomly.ab. 26 trial.ab. 27 groups.ab. 28 or/20–27 29 exp animals/ not humans.sh. 30 28 not 29

**Table 2:** Eletronic search strategies presented to the main databases.

(Cochrane library)
[mh ^“Posterior Cruciate Ligament”] ((posterior:ti,ab AND cruciate:ti,ab AND ligament:ti,ab) OR PCL:ti,ab) #1 OR #2 [mh “Surgical Procedures, Operative”] [mh “Orthopedic Fixation Devices”] [mh ^”Orthopedic Procedures”] (surg*:ti,ab OR operat*:ti,ab OR reconstruct*:ti,ab OR replace*:ti,ab OR repair*:ti,ab OR graft*:ti,ab) [mh/SU] #4 OR #5 OR #6 OR #7 OR #8 [mh “Physical Therapy Modalities”] [mh Rehabilitation] [mh Immobilization] [mh “Anti-Inflammatory Agents”] [mh ^”Conservative Treatment”] [mh ^Braces] OR [mh ^Crutches] (non-surg*:ti,ab OR nonsurg*:ti,ab OR non-operat*:ti,ab OR nonoperat*:ti,ab OR conserv*:ti,ab OR rehab*:ti,ab OR physiotherapy:ti,ab OR “physical therapy”:ti,ab OR exercis*:ti,ab OR brace*:ti,ab OR cast*:ti,ab OR plaster:ti,ab OR crutches:ti,ab OR immobli*:ti,ab OR anti-inflammatory:ti,ab OR non-steriod:ti,ab) #10 OR #11 OR #12 OR #13 OR #14 OR #15 OR #16 #9 OR #17 #3 AND #18 ”randomized controlled trial”:pt ”controlled clinical trial”:pt Randomi?Ed:ab Placebo:ab [mh/DT] Randomly:ab Trial:ab Groups:ab #20 OR #21 OR #22 OR #23 OR #24 OR #25 OR #26 OR #27 [mh animals] NOT [mh ^humans] #28 NOT #29 **(EMBASE)** “Posterior Cruciate Ligament”/ ((posterior AND cruciate AND ligament) OR PCL).tw. 1 OR 2 Exp “Surgical Procedures, Operative”/ Exp “Orthopedic Fixation Devices”/ ”Orthopedic Procedures”/ (surg* OR operat* OR reconstruct*OR replace* OR repair* OR graft*).tw. ”Surgery”.fs. 4 OR 5 OR 6 OR 7 OR 8 Exp “Physical Therapy Modalities”/ Exp Rehabilitation/ Exp Immobilization/ Exp “Anti-Inflammatory Agents”/ ”Conservative Treatment”/ Braces/ OR Crutches/ (non-surg*ornonsurg*OR non-operat*OR nonoperat*OR conserv*OR rehab* OR physiotherapy OR”physical therapy”OR exercis*OR brace* OR cast*OR plaster OR crutches OR immobli*OR anti-inflammatory OR non-steriod).tw. 10 OR 11 OR 12 OR 13 OR 14 OR 15 OR 16 9 OR 17 3 AND 18 “randomized controlled trial”.pt. ”controlled clinical trial”.pt. Randomi?Ed.ab. Placebo.ab.
”Drug Therapy”.fs. Randomly.ab. Trial.ab. Groups.ab. 20 OR 21 OR 22 OR 23 OR 24 OR 25 OR 26 OR 27 Exp animals/ NOT humans/ 28 NOT 29

**Table 3:** Eletronic search strategies presented to the main databases.

(LILACS)
1 (“ligamento cruzado posterior”) 2 (ligamento AND cruzado AND posterior) OR PCL 3 1 OR 2 4 (“procedimientos quirúrgicos”) 5 (“fijacion ortopédica”) 6 (“procedimientos ortopédicos”) 7 (cirúrg* OR operador* OR reconstr* OR substitu* OR repar* OR enxert*) 8 (“procedimientos quirúrgicos”[decs]) 9 4 OR 5 OR 6 OR 7 OR 8 10 (“fisioterapia”) 11 (“rehabilitación”) 12 (“inmovilización”) 13 (“agentes antiinflamatorios”) 14 (“tratamento conservador”) 15 (brac* OR muleta*) 16 (conservative OR nonsurgical OR nonoperativa OR physiotherapy OR physical OR exerc* OR brace OR plaster OR crutches OR immobi* OR non-steroidal OR rehab*) 17 10 OR 11 OR 12 OR 13 OR 14 OR 15 OR 16 18 9 OR 17 19 3 AND 18 20 (ensayo clínico controlado OR ensayo controlado) 21 (aleatorizado OR randomized) 22 (placebo) 23 (terapia) 24 (drug OR fármaco) 25 (ensayo OR trial) 26 (grupos OR groups) 27 20 OR 21 OR 22 OR 23 OR 24 OR 25 OR 26 28 (animal OR animales) NOT (humano OR humanos) 29 27 NOT 28

### Study selection

Two authors will screen abstracts and titles for inclusion of all the potentially relevant trials identified as a result of the search and code them as ‘retrieve’ (eligible, potentially eligible, or unclear) or not retrieved. The authors will retrieve the full text of the study reports or publications, and two review authors will independently screen the full text to identify trials for inclusion and record reasons for exclusion of the ineligible trials. Disagreements will be resolved by discussion and, if necessary, by a third author [5,21].

**Fig 1:**
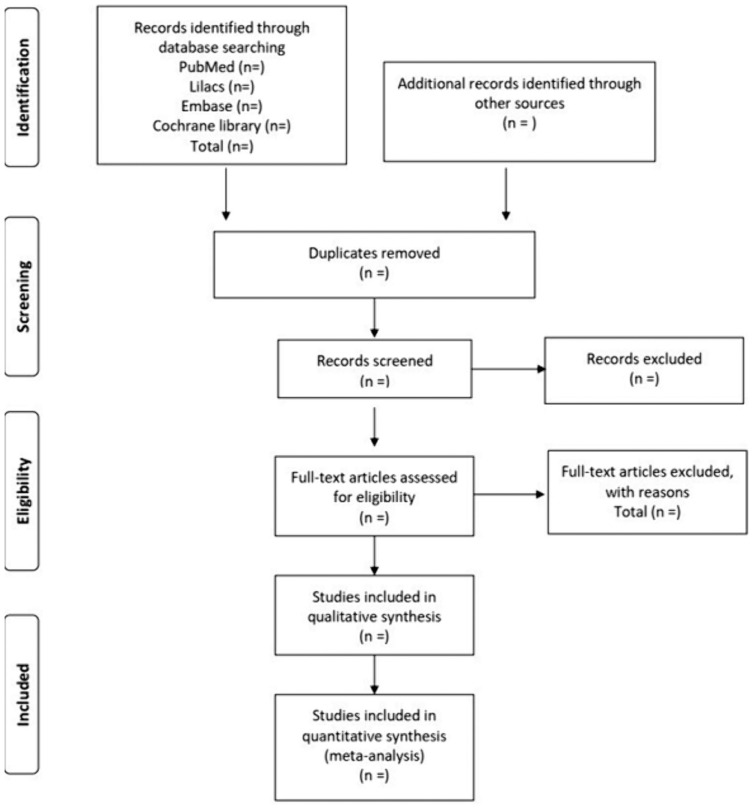
Preferred Reporting Items for Systematic Reviews and Meta-Analyses flow chart.

The Rayyan software (www.rayyan.ai) will be used to manage the study selection process, particulary for duplicate removal and screening.The review authors will use an Excel spreadsheet to record the decision-making, containing an explanation regarding the inclusion or exclusion of the randomized trial. If any study is incomplete regarding the necessary information, they will contact the author by email for clarification, and if the original author does not reply, they will label the trial as ‘missing information’ [21].The minutiae of the full selection action are shown in the PRISMA flow chart in Fig 1 [21].

### Data management

All the extracted data will be stored and accessed in Rayyan software and in Review Manager V.5.4/Review Manager Web software [34].

### Data extraction

The analysis will be conducted using Microsoft Excel (Microsoft Corporation, Redmond, Washington, USA). Two review authors will independently perform data extraction using a data collection form piloted on all included studies. The authors will independently extract the following data from each eligible trial [5,21]:

Methods: Study design, study duration, study locations, study setting, date of study and funding of the study.

Characteristics of the population (participants): sample size (N), number randomized, number lost (to follow-up and/or withdrawn), age range, mean age, sex, severity of the condition, diagnostic criteria, inclusion and exclusion criteria, unilateral or bilateral injury, classification of the PCL lesion, partial or complete lesion, and presence or absence of concomitant lesions.

Intervention: all details of any intervention (nonsurgical or surgical) and its comparison.

Outcomes (including measures and time points).

Numerical data for outcomes of interest.

Type of analysis/analyses presented.

Notes: notable declarations of interest of trial authors and funding for trials. Details will be obtain if it was possible to contact the authors of the trials.

Information needed to assess bias (e.g., presence or absence of a protocol, a new or old device or technique, deviations from intended interventions, use of data imputed for key outcomes, etc.).

Information needed to assess the Grading of Recommendations, Assessment, Development and Evaluation (GRADE) (e.g., baseline risk in the control group for key outcomes, heterogeneity, indirectness, etc.).

Disagreements will be resolved by a third author [5,21].

### Risk of bias assessment

Two authors will independently assess the risk of bias of the included studies using the Risk of Bias 2 tool (a revised Cochrane risk-of-bias tool for randomized trials) [21]. The risk of bias will be assessed for all predefined primary and secondary outcomes [33,35]. The following five methodological domains will be evaluated for risk of bias: (1) arising from the randomization process, (2) due to deviations from the intended interventions, (3) due to missing outcome data, (4) in measurement of the outcome and (5) in selection of the reported result [21].

A ‘low’ risk of bias, ‘high’ risk of bias or ‘some concerns' judgement will be assigned to each domain; the last reflecting an absence of information or uncertainty about the potential for bias. Any disagreements between the review authors with reference to the risk of bias for each domain will be settled by discussion or by a third author, if necessary [21,36–38].

### Missing data

The authors will perform an intention-to-treat analysis to include all randomized participants of any intervention. The authors of the selected trials will be contacted regarding insufficient information according to the estimated effects as well as the number of participants, the number of events and uncertainty in the measurements. The analysis will be performed independently of the lost data according to the best-case and worst-case scenarios [5,33,39,37].

### Descriptive analysis

The authors will describe all included trials in detail, with a valid tool because of heterogeneous information, inclusion criteria, data collection methods, varied objectives, participant characteristics, different outcomes, different interventions and others [5].

### Statistical analyses

#### Measures of treatment effect.

The authors will express dichotomous data as risk ratios (RRs) with 95% confidence intervals (CIs). For continuous outcomes measured on the same scale (e.g., body weight in kg), the intervention effect will be estimated using the mean difference (MD) with the corresponding standard deviation (SD) and 95% CIs. For continuous outcomes that measure the same underlying concept (e.g., knee functional score, health‐related quality of life) but use different measurement scales, the standardized mean difference (SMD) will be calculated. The magnitude of the SMD will be interpreted according to Cohen (small/minor SMD: 0.2 or less, medium SMD: 0.2 to 0.8, large SMD: 0.8 or greater) [5,21,34,38,40].

**Unit of analysis issues:** The unit of randomization for the included studies is likely to be individual participants. When studies included patients with bilateral posterior cruciate ligament lesions, the data may be evaluated for lesions rather than for individual patients. When such a unit of analysis issues arises and appropriate corrections have not been made, the authors will consider presenting data for such trials only where the disparity between the unit of analysis and randomization is small. The authors will not include crossover or cluster-randomized trials in this review [5].

#### Assessment of heterogeneity.

The heterogeneity of estimate effects between the included trials will be assessed by visual inspection of the forest plot (analysis) along with consideration of the chi-squared test for heterogeneity and the I-squared statistic. The authors will define heterogeneity based on I^2^ values as follows: 25%, low heterogeneity; 50%, moderate heterogeneity; and 90%, high heterogeneity. All the statistical analyses will be performed using Review Manager V.5.4 software, with p value of <0.05 considered statistically significant [5,21].

#### Assessment of reporting biases.

The review authors will assess publication bias by visually checking funnel plot asymmetry where sufficient trials and data are available [36,37]. The authors will create a funnel plot to explore possible small study biases if there are at least 10 trials included in the meta‐analysis. The funnel plots will be interpreted to determine the possible reasons for funnel plot asymmetry, as described in Chapter 13 of the *Cochrane Handbook for Systematic Reviews of Interventions.* If the authors are able to pool more than 10 trials, they will undertake formal statistical tests to investigate funnel plot asymmetry and will follow the recommendations in chapter 13.3 of the *Cochrane Handbook for Systematic Reviews of Interventions* [37,38].

To assess outcome reporting bias, the trial protocols will be checked against published reports. The authors will evaluate whether selective reporting of outcomes is present, too [5,36,37].

#### Subgroup analysis.

The authors will investigate surgical and nonsurgical management of posterior cruciate ligament injuries.

The following data will be analyzed: type of posterior cruciate ligament injury (total or partial, acute or chronic), mechanism of injury (caused by high energy or low energy), age of the patients, type (technique) of the surgery or conservative treatment, whether the PCL lesion or injury was partial or complete, and whether the injury was isolated or concomitant.

The formal test for subgroup intersection will be used in Review Manager 5.4/Rev.Man. The review authors will use Revman web software and interpret the results as advised in chapter 10.11.2 of the *Cochrane Handbook for Systematic Reviews of Interventions*. The authors will compare the magnitude of the effect between subgroups by assessing the overlap between CIs (interval confidence) of the summary effect estimates, where non‐overlapping CIs indicate statistical significance [5].

### Data synthesis

#### Strategy for data synthesis.

The results of comparable groups of trials will be pooled when possible and appropriate (if participants, interventions, comparisons and outcomes are sufficiently similar in the included trials). The authors will conduct all meta‐analyses using Review Manager 5.4/Rev.Man. Web software. It is anticipated that the underlying effect of the intervention may vary between studies, as there are likely differences between participants, settings and the interventions used for each study. Therefore, the authors will use a random‐effects method for meta‐analysis [34,40,41].

Estimation effects will be expressed as the number needed to treat (NNT) when appropriate.

For dichotomous data, treatment differences will be analyzed as a risk ratio (RR) calculated using the Mantel‐Haenszel method [5].

For continuous outcomes, if all data was from the same scale, the mean follow‐up values will be pooled with change‐from‐baseline data, and the results will be reported as the mean difference. If there is a need to report standardized mean differences, then the review authors will not pool endpoint and change‐from‐baseline data [5].

If the included studies do not allow pooling of data, the review authors will present the results in a narrative format [40–42].

#### Sensitivity analysis.

The review authors plan to carry out the following sensitivity analyses for the main comparisons to investigate the robustness of the treatment effect on critical outcomes:

Impact of including studies with high or unclear risk of detection, selection, and attrition biases.

Impact of including studies with imputed data.

Impact of excluding trials with a high risk of bias or those with small sample sizes.

The Grading of Recommendations, Assessment, Development and Evaluation (GRADE) approach will be used to assess the quality of evidence related to each of the primary outcomes listed [5,43,44].

#### Confidence in cumulative evidence.

The quality of evidence for each outcome will be assessed using GRADE [42–45]. The quality of the studies outcomes will be classified into four categories: high, moderate, low or very low. The authors will create “Summary of Finding Table” for the following outcomes: 1-Validated health-related quality of life scores. 2-Validated patient-rated measures of knee function. 3- Pain: [5,46–49].

The authors will use the methods and recommendations described in chapter 12 of the Cochrane Handbook for Systematic Reviews of Interventions, using GRADE Pro software [35,39,37,44,48–50].

#### Patient consent for publication.

Not applicable.

## Discussion

### Expected benefits

Many patients have cruciate ligament injuries of the knee nowadays. This systematic review and meta-analysis will help Orthopaedic surgeons, Knee surgeons and patients to make more informed decisions.

### Future research directions

The authors will carry out a Future Research Directions after analyzing the results found in this review. It will identify gaps in the literature that need to be addressed in subsequent studies. Therefore, the authors can point out the flaws and gaps of the existing literature and indicate the best study designs that can be carried out in the future.

### Strengths and limitations of this study

This systematic review protocol follows the guidelines of the Cochrane Handbook, PRISMA and is registered in PROSPERO.

The strength of this systematic review is based on the fact that there will be no restriction in the studies`selection based on the publication date and/or language.

This review will only include randomized clinical trials and will be evaluated using the main databases, resulting in a better level of evidence.

It will be difficult to pool data with various interventions for treating PCL injuries and we anticipate a high degree of heterogeneity in the included trials, so subgroup analyses will be conducted to address this problem.

## Conclusion

This systematic review will be a comprehensive assessment of current state of evidence on the effectiveness of the interventions (surgical and conservative) for treating PCL injuries of the knee in adults, will build a foundation for informed health decision-making and will help to identify potential areas of improvement in research and clinical areas.

## Supporting information

S1 File PRISMA-P checklist. (DOCX)

## References

[1] Amis AA, Gupte CM, Bull AMJ, Edwards A. Anatomy of the posterior cruciate ligament and the meniscofemoral ligaments. Knee Surg Sports Traumatol Arthrosc. 2006;14(3):257–63. doi: 10.1007/s00167-005-0686-x 16228178

[2] Anderson JE. Grant’s atlas of anatomy. 7th ed. Vol. 1. Baltimore: Williams and Wilkins; 1978. pp. 576–92.

[3] Insall JN, Scott WN. Surgery of the knee. 4 ed. Vol. 2. Elsevier; 2006; pp. 1205–11.

[4] Nicolini AP, de Carvalho RT, Matsuda MM, Sayum JF, Cohen M. Common injuries in athletes’ knee: experience of a specialized center. Acta Ortop Bras. 2014;22(3):127–31. doi: 10.1590/1413-78522014220300475 25061417 PMC4108693

[5] Sayum Filho J, Lenza M, Teixeira de Carvalho R, Pires OGN, Cohen M, Belloti JC. Interventions for treating fractures of the patella in adults. Cochrane Database Syst Rev. 2015;(2):CD009651. doi: 10.1002/14651858.CD009651.pub2 25723760

[6] Butler DL, Noyes FR, Grood ES. Ligamentous restraints to anterior-posterior drawer in the human knee. A biomechanical study. J Bone Joint Surg Am. 1980;62(2):259–70. 7358757

[7] Fanelli G. Posterior cruciate ligament. Sports Med Arthrosc Rev. 2020;28(1):1–10. 31895323 10.1097/JSA.0000000000000280

[8] Yoon KH, Bae DK, Song SJ, Cho HJ, Lee JH. A prospective randomized study comparing arthroscopic single-bundle and double-bundle posterior cruciate ligament reconstructions preserving remnant fibers. Am J Sports Med. 2011;39(3):474–80. doi: 10.1177/0363546510382206 21098819

[9] Rubinstein RA Jr, Shelbourne KD, McCarroll JR, VanMeter CD, Rettig AC. The accuracy of the clinical examination in the setting of posterior cruciate ligament injuries. Am J Sports Med. 1994;22(4):550–7. doi: 10.1177/036354659402200419 7943523

[10] Umile GL, Marco V, Vincenzo C, Girolamo L, Cella E. Epidemiology of posterior cruciate ligament reconstructions in Italy: a 15-year study. J Clin Med. 2021;10(3):499–502. 33535403 10.3390/jcm10030499 PMC7867089

[11] Li J, Kong F, Gao X, Shen Y, Gao S. Prospective randomized comparison of knee stability and proprioception for posterior cruciate ligament reconstruction with autograft, hybrid graft, and γ-irradiated allograft. Arthroscopy. 2016;32(12):2548–55. doi: 10.1016/j.arthro.2016.04.024 27282110

[12] Qi Y-S, Wang H-J, Wang S-J, Zhang Z-Z, Huang A-B, Yu J-K. A systematic review of double-bundle versus single-bundle posterior cruciate ligament reconstruction. BMC Musculoskelet Disord. 2016;17:45. doi: 10.1186/s12891-016-0896-z 26818255 PMC4730768

[13] Migliorini F, Pintore A, Vecchio G, Oliva F, Hildebrand F, Maffulli N. Hamstring, bone-patellar tendon-bone, quadriceps and peroneus longus tendon autografts for primary isolated posterior cruciate ligament reconstruction: a systematic review. Br Med Bull. 2022;142(1):23–33. doi: 10.1093/bmb/ldac010 35460407 PMC9351477

[14] Krott NL, Wengle L, Whelan D, Wild M, Betsch M. Single and double bundle posterior cruciate ligament reconstruction yield comparable clinical and functional outcomes: a systematic review and meta-analysis. Knee Surg Sports Traumatol Arthrosc. 2022;30(7):2388–99. doi: 10.1007/s00167-022-06907-6 35174403

[15] Schroven W, Vles G, Verhaegen J, Roussot M, Bellemans J, Konan S. Operative management of isolated posterior cruciate ligament injuries improves stability and reduces the incidence of secondary osteoarthritis: a systematic review. Knee Surg Sports Traumatol Arthrosc. 2022;30(5):1733–43. doi: 10.1007/s00167-021-06723-4 34505176

[16] Zhao J, Huang-Fu X, He Y, Yang X. Single-bundle posterior cruciate ligament reconstruction with remnant preservation: lateral versus medial-sided augmentation technique. Orthop Surg. 2009;1(1):66–73. doi: 10.1111/j.1757-7861.2008.00012.x 22009784 PMC6734644

[17] Li Y, Li J, Wang J, Gao S, Zhang Y. Comparison of single-bundle and double-bundle isolated posterior cruciate ligament reconstruction with allograft: a prospective, randomized study. Arthroscopy. 2014;30(6):695–700. doi: 10.1016/j.arthro.2014.02.035 24731384

[18] Fischer SP, Fox JM, Del Pizzo W, Friedman MJ, Snyder SJ, Ferkel RD. Accuracy of diagnoses from magnetic resonance imaging of the knee. A multi-center analysis of one thousand and fourteen patients. J Bone Joint Surg Am. 1991;73(1):2–10. 1985991

[19] DeHaven KE, Cosgarea AJ, Sebastianelli WJ. Arthrofibrosis of the knee following ligament surgery. Instr Course Lect. 2003;52:369–81. 12690864

[20] Yoon KH, Park SW, Lee SH, Kim MH, Park SY, Oh H. Does cast immobilization contribute to posterior stability after posterior cruciate ligament reconstruction? Arthroscopy. 2013;29(3):500–6. doi: 10.1016/j.arthro.2012.10.019 23351730

[21] Tossolini Goulart L, Matsunaga FT, Belloti JC, Faloppa F, Paim TS, Tamaoki MJS. Effectiveness of subacromial injections in rotator cuff lesions: systematic review and meta-analysis protocol. BMJ Open. 2022;12(11):e062114. doi: 10.1136/bmjopen-2022-062114 36323483 PMC9639075

[22] Sousa TS, Jardim RAC, Silva CF, Sousa AS, Iosimuta N, Trevisani VF, et al. Early mobilization after skin graft for burn injury in adults. Cochrane Database Syst Rev. 2025;5(5):CD016109. doi: 10.1002/14651858.CD016109 40365848 PMC12076552

[23] Liberati A, Altman DG, Tetzlaff J, Mulrow C, Gøtzsche PC, Ioannidis JPA, et al. The PRISMA statement for reporting systematic reviews and meta-analyses of studies that evaluate health care interventions: explanation and elaboration. PLoS Med. 2009;6(7):e1000100. doi: 10.1371/journal.pmed.1000100 19621070 PMC2707010

[24] Page MJ, McKenzie JE, Bossuyt PM, Boutron I, Hoffmann TC, Mulrow CD, et al. The PRISMA 2020 statement: an updated guideline for reporting systematic reviews. BMJ. 2021;372:n71. doi: 10.1136/bmj.n71 33782057 PMC8005924

[25] Cumpston M, Li T, Page MJ, Chandler J, Welch VA, Higgins JPT, et al. Cochrane Database of Systematic Reviews. Cochrane Library; 2019. 10.1002/14651858.ED000142 PMC10284251 31643080

[26] Cumpston M, Lasserson T, Chandler J, Page MJ. Chapter III: Reporting the review. In: Higgins JP, Thomas J, Chandler J, Cumpston M, Li T, Page MJ, Welch VA, editors. Cochrane Handbook for Systematic Reviews of Interventions Version 6.3. Cochrane; 2022. www.training.cochrane.org/handbook

[27] Tegner Y, Lysholm J. Rating systems in the evaluation of knee ligament injuries. Clin Orthop Relat Res. 1985;(198):43–9. 4028566

[28] Ware JE Jr, Sherbourne CD. The MOS 36-item short-form health survey (SF-36). I. Conceptual framework and item selection. Med Care. 1992;30(6):473–83. 1593914

[29] Lysholm J, Gillquist J. Evaluation of knee ligament surgery results with special emphasis on use of a scoring scale. Am J Sports Med. 1982;10(3):150–4. doi: 10.1177/036354658201000306 6896798

[30] Marshall JL, Fetto JF, Botero PM. Knee ligament injuries: a standardized evaluation method. Clin Orthop Relat Res. 1977;123:115–29. 856512

[31] Roos EM, Roos HP, Lohmander LS, Ekdahl C, Beynnon BD. Knee Injury and Osteoarthritis Outcome Score (KOOS)--development of a self-administered outcome measure. J Orthop Sports Phys Ther. 1998;28(2):88–96. doi: 10.2519/jospt.1998.28.2.88 9699158

[32] Lefebvre C, Manheimer E, Glanville J. Chapter 6: Searching for studies. In: Higgins JPT, Green S, editors. Cochrane Handbook for Systematic Reviews of Interventions Version 5.1.0. The Cochrane Collaboration; 2011. www.cochrane-handbook.org

[33] Lefebvre C, Glanville J, Briscoe S, Featherstone R, Littlewood A, Marshall C, et al. Technical Supplement to Chapter 4: Searching for and selecting studies. In: Higgins JP, Thomas J, Chandler J, Cumpston MS, Li T, Page MJ, Welch VA, editors. Cochrane Handbook for Systematic Reviews of Interventions Version 6.3. Cochrane, 2022. www.training.cochrane.org/handbook

[34] Ouzzani M, Hammady H, Fedorowicz Z, Elmagarmid A. Rayyan-a web and mobile app for systematic reviews. Syst Rev. 2016;5(1):210. doi: 10.1186/s13643-016-0384-4 27919275 PMC5139140

[35] Higgins JPT, Altman DG, Sterne JAC. Chapter 8: Assessing risk of bias in included studies. In: Higgins JPT, Green S, editors. Cochrane Handbook for Systematic Reviews of Interventions Version 5.1.0. The Cochrane Collaboration; 2011.

[36] Sterne JAC, Savović J, Page MJ, Elbers RG, Blencowe NS, Boutron I, et al. RoB 2: a revised tool for assessing risk of bias in randomised trials. BMJ. 2019;366:l4898. doi: 10.1136/bmj.l4898 31462531

[37] Higgins JPT, Li T, Deeks JJ. Chapter 6: Choosing effect measures and computing estimates of effect. In: Higgins JPT, Thomas J, Chandler J, Cumpston M, Li T, Page MJ, et al., editors. Cochrane Handbook for Systematic Reviews of Interventions Version 6.2. Cochrane; 2021. www.training.cochrane.org/handbook/archive/v6.2/

[38] Higgins JP, Li T, Deeks JJ. Chapter 6: Choosing effect measures and computing estimates of effect. In: Higgins JP, Thomas J, Chandler J, Cumpston M, Li T, Page MJ, Welch VA, editors. Cochrane Handbook for Systematic Reviews of Interventions Version 6.3. 2022. www.training.cochrane.org/handbook

[39] Balshem H, Helfand M, Schünemann HJ, Oxman AD, Kunz R, Brozek J, et al. GRADE guidelines: 3. Rating the quality of evidence. J Clin Epidemiol. 2011;64(4):401–6. doi: 10.1016/j.jclinepi.2010.07.015 21208779

[40] Review Manager Web (RevMan Web) [Computer program]. Version 4.6.0. The Cochrane Collaboration. 2022. Available from: revman.cochrane.org

[41] Deeks JJ, Higgins JP, Altman DG. Chapter 10: Analysing data and undertaking meta-analyses. In: Higgins JP, Thomas J, Chandler J, Cumpston M, Li T, Page MJ, Welch VA, editors. Cochrane Handbook for Systematic Reviews of Interventions Version 6.3. Cochrane; 2022. www.training.cochrane.org/handbook

[42] Egger M, Davey Smith G, Schneider M, Minder C. Bias in meta-analysis detected by a simple, graphical test. BMJ. 1997;315(7109):629–34. doi: 10.1136/bmj.315.7109.629 9310563 PMC2127453

[43] GRADEpro GDT [Computer program]. Hamilton (ON): McMaster University (developed by Evidence Prime). [cited 27 Apr 2022]. Available from: gradepro.org

[44] Guyatt GH, Oxman AD, Vist G, Kunz R, Brozek J, Alonso-Coello P, et al. GRADE guidelines: 4. Rating the quality of evidence--study limitations (risk of bias). J Clin Epidemiol. 2011;64(4):407–15. doi: 10.1016/j.jclinepi.2010.07.017 21247734

[45] Langer G, Meerpohl JJ, Perleth M, Gartlehner G, Kaminski-Hartenthaler A, Schünemann H. GRADE guidelines: 1. Introduction - GRADE evidence profiles and summary of findings tables. Z Evid Fortbild Qual Gesundhwes. 2012;106(5):357–68. doi: 10.1016/j.zefq.2012.05.017 22818160

[46] Langer G, Meerpohl JJ, Perleth M, Gartlehner G, Kaminski-Hartenthaler A, Schünemann H. GRADE guidelines: 2. Framing the question and deciding on important outcomes. Z Evid Fortbild Qual Gesundhwes. 2012;106(5):369–76. 22818161 10.1016/j.zefq.2012.05.018

[47] Schunemann HJ, Oxman AD, Vist GE, Higgins JPT, Deeks JJ, Glaziou P, et al. Chapter 12: Interpreting results and drawing conclusions. In: Higgins JPT, Green S, editors. Cochrane Handbook for Systematic Reviews of Interventions Version 5.1.0. The Cochrane Collaboration; 2011. www.cochrane-handbook.org

[48] Schünemann HJ, Higgins JP, Vist GE, Glasziou P, Akl EA, Skoetz N, et al. Chapter 14: Completing ‘Summary of findings’ tables and grading the certainty of the evidence. In: Higgins JP, Thomas J, Chandler J, Cumpston M, Li T, Page MJ, Welch VA, editors. Cochrane Handbook for Systematic Reviews of Interventions Version 6.3. Cochrane; 2022. www.training.cochrane.org/handbook

[49] Hasford J, Bramlage P, Koch G, Lehmacher W, Einhäupl K, Rothwell PM. Standards for subgroup analyses are needed?--we couldn’t agree more. J Clin Epidemiol. 2011;64(4):451; author reply 452. doi: 10.1016/j.jclinepi.2010.10.001 21227650

[50] Cohen J. Statistical power analysis for the behavioral sciences. 2nd ed. Hillsdale, NJ: Lawrence Erlbaum Associates; 1988.

