# Supplementary material for: Interventions for treating posterior cruciate ligament injuries of the knee in adults: A systematic review and meta-analysis protocol
Source: PLoS One. 2025 Sep 25;20(9):e0333015. doi: 10.1371/journal.pone.0333015 (PMC12463263; doi:10.1371/journal.pone.0333015)
Supplement: S1 File — (DOCX) [file pone.0333015.s001.docx]

PRISMA-P (Preferred Reporting Items for Systematic review and Meta-Analysis Protocols) 2015 checklist: recommended items to address in a systematic review protocol*

| **Section and topic** | **Item No** | | **Checklist item** |
| --- | --- | --- | --- |
| **ADMINISTRATIVE INFORMATION** | | | |
| Title: |  |  | |
| Identification | 1a | Identify the report as a protocol of a systematic review **Interventions for treating posterior cruciate ligament injuries of the knee in adults: a systematic review protocol.** | |
| Update | 1b | If the protocol is for an update of a previous systematic review, identify as such It is not | |
| Registration | 2 | If registered, provide the name of the registry (such as PROSPERO) and registration number It was registered in Prospero | |
|  |  | **registration number** CRD42023430493. | |
| Authors: |  |  | |
| Contact | 3a | Provide name, institutional affiliation, e-mail address of all protocol authors; provide physical mailing address of corresponding author  **Interventions for treating posterior cruciate ligament injuries of the knee in adults: a systematic review protocol. Jorge Sayum Filho [1;2;3]; Marcel JS Tamaoki [1;3]; Rogerio Teixeira de Carvalho[1;3]; Álvaro N Atallah [2;3]; Fabio T Matsunaga[1;3]; Flávio Faloppa[1;3]; João Carlos Belloti[1;3].**  **[1] Department of Orthopaedics and Traumatology, Universidade Federal de São Paulo, São Paulo, Brazil [2] Cochrane Brazil, Centro de Estudos de Saúde Baseada em Evidências e Avaliação Tecnológica em Saúde, São Paulo, Brazil. [3] PPG Cirurgia Translacional - Universidade Federal de São Paulo, São Paulo, Brazil**  **Correspondence to Dr. Jorge Sayum Filho:** [**jorgesayumfilho@hotmail.com**](mailto:jorgesayumfilho@hotmail.com)  **Marcel Jun Sugawara Tamaoki: marceltamaoki@gmail.com**  **Rogerio Teixeira de Carvalho: rtcarv@terra.com.br**  **Álvaro Nagib Atallah: Álvaro.atallah@gmail.com**  **Fabio Teruo Matsunaga:fteruo@gmail.com**  **Flávio Faloppa: faloppa.dot@gmail.com**  **João Carlos Belloti: jcbelloti@gmail.com** | |
| Contributions | 3b | Describe contributions of protocol authors and identify the guarantor of the review Authors’ contributions  - **Contributors JSF—submitting author, guarantor and corresponding author. RT, FTM, JCB, FF, ANA and MJST—authorship.JSF, RT and MJST devised the project and collected data in the literature about the content covered. MJST, ANA and FF conceived the study and was in charge of the overall direction. FTM and JCB structured the manuscript according to scientific norms. RT, JCB and JSF contributed to the study design and wrote the manuscript. They will evaluate the data and formulate the planned meta-analyses. JCB and FF reviewed the study proposal and contributed to the implementation of the research.** | |
| Amendments | 4 | If the protocol represents an amendment of a previously completed or published protocol, identify as such and list changes; otherwise, state plan for documenting important protocol amendments. **Our plan for documenting amendments is that there will be a chapter called differences between the systematic review and the protocol in the review**. | |
| Support: |  |  | |
| Sources | 5a | Indicate sources of financial or other support for the review. No sources | |
| Sponsor | 5b | Provide name for the review funder and/or sponsor. No funder or sponsor | |
| Role of sponsor or funder | 5c | Describe roles of funder(s), sponsor(s), and/or institution(s), if any, in developing the protocol. The authors thank the Universidade Federal De São Paulo for providing the structure for the realization of this review | |
| **INTRODUCTION** |  |  | |
| Rationale | 6 | Describe the rationale for the review in the context of what is already known  **An explanation of why our manuscript should be published**  Interventions for treating posterior cruciate ligament injuries of the knee in adults. It is an interesting topical. The tear of the PCL is reported to represent 0.65 to 3 % of the total sports related knee injuries . Occurs more in young male adults. The PCL tears represent 20% of all knee ligament injuries. In the last years, greater participation in sporting and recreational activities by the general population, in all around the world, continues to expose more individuals to the risk of posterior cruciate ligament lesion. PCL injuries can be treated conservatively or surgically. There are many surgical and conservative techniques for treating the PCL injuries, but there is no answer for the best method or technique.  About this topic, in pubmed we found at least 9 randomised controlled trials:  Orthop Surg. 2009 Feb;1(1):66-73. doi: 10.1111/j.1757-7861.2008.00012.x.Singlebundle posterior cruciate ligament reconstruction with remnant preservation: lateral versus medial-sided augmentation technique. Jin-zhong Zhao 1, Xiao-qiao Huang-Fu, Yao-hua He, Xing-guang Yang.PMID: 22009784. PMCID: PMC6734644.DOI: 10.1111/j.1757-7861.2008.00012.x.  Am J Sports Med. 2011 Mar;39(3):474-80. doi: 10.1177/0363546510382206. Epub 2010 Nov 23.A prospective randomized study comparing arthroscopic single-bundle and double-bundle posterior cruciate ligament reconstructions preserving remnant fibers. Kyoung Ho Yoon 1, Dae Kyung Bae, Sang Jun Song, Hyung Jun Cho, Jung Hwan Lee. PMID: 21098819. DOI:  Arthroscopy. 2014 Jun;30(6):695-700. doi: 10.1016/j.arthro.2014.02.035. Epub 2014 Apr 14.Comparison of single-bundle and double-bundle isolated posterior cruciate ligament reconstruction with allograft: a prospective, randomized study. Yongqian Li 1, Jia Li 1, Jianzhao Wang 1, Shijun Gao 1, Yingze Zhang 2.PMID: 24731384.DOI: 10.1016/j.arthro.2014.02.035  Arthroscopy. 2016 Dec;32(12):2548-2555. doi: 10.1016/j.arthro.2016.04.024. Epub 2016 Jun 7Prospective Randomized Comparison of Knee Stability and Proprioception for Posterior Cruciate Ligament Reconstruction With Autograft, Hybrid Graft, and γIrradiated Allograft. Jia Li 1, Fanlong Kong 1, Xianda Gao 1, Yong Shen 1, Shijun Gao 2, PMID: 27282110. DOI: 10.1016/j.arthro.2016.04.024. 10.1177/0363546510382206  Arthroscopy. 2013 Mar;29(3):500-6. doi: 10.1016/j.arthro.2012.10.019. Epub 2013 Jan 23. Does cast immobilization contribute to posterior stability after posterior cruciate ligament reconstruction? Kyoung Ho Yoon 1, Sung Woo Park, Sang Hak Lee, Man Ho Kim, Soo Yeon Park, Hoon Oh. PMID: 23351730.DOI: 10.1016/j.arthro.2012.10.019.  Zhongguo Xiu Fu Chong Jian Wai Ke Za Zhi. . 2020 Jun 15;34(6):713-719. doi: 10.7507/1002-1892.201910115.[Clinical study on reconstruction of posterior cruciate ligament with platelet rich plasma combined with 3-strand peroneus longus tendons] [Article in Chinese].[Shichun Wu](https://pubmed.ncbi.nlm.nih.gov/?term=Wu+S&cauthor_id=32538561) [^1^](https://pubmed.ncbi.nlm.nih.gov/32538561/#full-view-affiliation-1), [Wenxiang Lin](https://pubmed.ncbi.nlm.nih.gov/?term=Lin+W&cauthor_id=32538561) [^1^](https://pubmed.ncbi.nlm.nih.gov/32538561/#full-view-affiliation-1), [Weihua Xu](https://pubmed.ncbi.nlm.nih.gov/?term=Xu+W&cauthor_id=32538561) [^1^](https://pubmed.ncbi.nlm.nih.gov/32538561/#full-view-affiliation-1), [Honghan Li](https://pubmed.ncbi.nlm.nih.gov/?term=Li+H&cauthor_id=32538561) [^1^](https://pubmed.ncbi.nlm.nih.gov/32538561/#full-view-affiliation-1) PMID: **32538561.** PMCID: [PMC8171524](http://www.ncbi.nlm.nih.gov/pmc/articles/pmc8171524/) DOI: [10.7507/1002-1892.201910115](https://doi.org/10.7507/1002-1892.201910115). Knee Surg Sports Traumatol Arthrosc. . 2015 Oct;23(10):3012-8.   doi: 10.1007/s00167-015-3719-0. Epub 2015 Aug 20. External fixator for treatment of the sub-acute and chronic multi-ligament-injured knee. [Fabio Janson Angelini](https://pubmed.ncbi.nlm.nih.gov/?term=Angelini+FJ&cauthor_id=26289092) [^1^](https://pubmed.ncbi.nlm.nih.gov/26289092/#full-view-affiliation-1), [Camilo Partezani Helito](https://pubmed.ncbi.nlm.nih.gov/?term=Helito+CP&cauthor_id=26289092) [^2^](https://pubmed.ncbi.nlm.nih.gov/26289092/#full-view-affiliation-2), [Marcelo Batista Bonadio](https://pubmed.ncbi.nlm.nih.gov/?term=Bonadio+MB&cauthor_id=26289092) [^2^](https://pubmed.ncbi.nlm.nih.gov/26289092/#full-view-affiliation-2), [Tales Molica Guimarães](https://pubmed.ncbi.nlm.nih.gov/?term=Guimar%C3%A3es+TM&cauthor_id=26289092) [^2^](https://pubmed.ncbi.nlm.nih.gov/26289092/#full-view-affiliation-2), [Ronald Bispo Barreto](https://pubmed.ncbi.nlm.nih.gov/?term=Barreto+RB&cauthor_id=26289092) [^2^](https://pubmed.ncbi.nlm.nih.gov/26289092/#full-view-affiliation-2), [José Ricardo Pécora](https://pubmed.ncbi.nlm.nih.gov/?term=P%C3%A9cora+JR&cauthor_id=26289092) [^2^](https://pubmed.ncbi.nlm.nih.gov/26289092/#full-view-affiliation-2), [Gilberto Luis Camanho](https://pubmed.ncbi.nlm.nih.gov/?term=Camanho+GL&cauthor_id=26289092) [^2^](https://pubmed.ncbi.nlm.nih.gov/26289092/#full-view-affiliation-2), [Roberto Freire da Mota E Albuquerque](https://pubmed.ncbi.nlm.nih.gov/?term=da+Mota+E+Albuquerque+RF&cauthor_id=26289092) [^2^](https://pubmed.ncbi.nlm.nih.gov/26289092/#full-view-affiliation-2)PMID: **26289092.** DOI: [10.1007/s00167-015-3719-0](https://doi.org/10.1007/s00167-015-3719-0).  Knee Surg Sports Traumatol Arthrosc. 2021 Apr;29(4):1 251-1257.  doi: 10.1007/s00167-020-06144-9. Epub 2020 Jul 25. Arthroscopic reduction and internal fixation (ARIF) versus open reduction internal fixation (ORIF) to elucidate the difference for tibial side PCL avulsion fixation: a randomized controlled trial (RCT). [Silvampatti Ramaswamy Sundararajan](https://pubmed.ncbi.nlm.nih.gov/?term=Sundararajan+SR&cauthor_id=32712683) [^1^](https://pubmed.ncbi.nlm.nih.gov/32712683/#full-view-affiliation-1), [Joseph Babu Joseph](https://pubmed.ncbi.nlm.nih.gov/?term=Joseph+JB&cauthor_id=32712683) [^2^](https://pubmed.ncbi.nlm.nih.gov/32712683/#full-view-affiliation-2), [Rajagopalakrishnan Ramakanth](https://pubmed.ncbi.nlm.nih.gov/?term=Ramakanth+R&cauthor_id=32712683) [^2^](https://pubmed.ncbi.nlm.nih.gov/32712683/#full-view-affiliation-2), [Amit Kumar Jha](https://pubmed.ncbi.nlm.nih.gov/?term=Jha+AK&cauthor_id=32712683) [^2^](https://pubmed.ncbi.nlm.nih.gov/32712683/#full-view-affiliation-2), [Shanmuganathan Rajasekaran](https://pubmed.ncbi.nlm.nih.gov/?term=Rajasekaran+S&cauthor_id=32712683) [^3^](https://pubmed.ncbi.nlm.nih.gov/32712683/#full-view-affiliation-3) PMID: **32712683.** DOI: [10.1007/s00167-020-06144-9](https://doi.org/10.1007/s00167-020-06144-9)  J Bone Joint Surg Am. 2014 Feb 5;96(3):184-91.   doi: 10.2106/JBJS.L.01603. Hinged external fixation in the treatment of knee dislocations: a prospective randomized study. [James P Stannard](https://pubmed.ncbi.nlm.nih.gov/?term=Stannard+JP&cauthor_id=24500579) [^1^](https://pubmed.ncbi.nlm.nih.gov/24500579/#full-view-affiliation-1), [Clayton W Nuelle](https://pubmed.ncbi.nlm.nih.gov/?term=Nuelle+CW&cauthor_id=24500579) [^1^](https://pubmed.ncbi.nlm.nih.gov/24500579/#full-view-affiliation-1), [Gerald McGwin](https://pubmed.ncbi.nlm.nih.gov/?term=McGwin+G&cauthor_id=24500579) [^2^](https://pubmed.ncbi.nlm.nih.gov/24500579/#full-view-affiliation-2), [David A Volgas](https://pubmed.ncbi.nlm.nih.gov/?term=Volgas+DA&cauthor_id=24500579) [^1^](https://pubmed.ncbi.nlm.nih.gov/24500579/#full-view-affiliation-1) , PMID: **24500579.** DOI: [10.2106/JBJS.L.01603](https://doi.org/10.2106/jbjs.l.01603) | |
| Objectives | 7 | Provide an explicit statement of the question(s) the review will address with reference to participants, interventions, comparators, and outcomes (PICO) The review will address the question: What is the effectiveness of the interventions (surgical and conservative) for treating PCL injuries of the knee in adults?Population The authors will include trials involving adults over 17 years of age who were diagnosed with posterior cruciate ligament injuries (unilateral or bilateral) of the knee confirmed by magnetic resonance (MR) imaging.  **Intervention**  Randomized clinical trials comparing any methods of operative and conservative (nonsurgical treatment) interventions and comparisons with each other for the treatment of PCL injuries in adults.  **Comparator**  Any surgical or non-surgical treatment used for treating PCL injuries or no treatment.  **Outcomes** *Primary outcomes:* 1-Validated health-related quality of life scores (36-item Short Form (SF-36); (Ware) or others). 2-Validated patient-rated measures of knee function (the Lysholm Knee Scale, Cincinnati Knee Rating Scale or others). 3- Pain^25, 26, 27, 28, 29^.  *Secondary outcomes:* 1- Observer-rated measures of knee function (Roos; Tegner, and others). 2- Knee strength and range of motion. 3- Patient satisfaction with treatment. 4-Infection. 5-Treatment failure. 6-Time of rehabilitation and time to return to previous activities (labour, sports and others). 7-Adverse outcomes: early complications (wound infection or dehiscence, arthrofibrosis, paraesthesia, pain, loss of knee movement, and others); short-term complications (skin problems, stiffness, hardware irritation and loosening, loss of knee movement); and late complications (arthrofibrosis, sensitive or painful wound site, hardware irritation or loosening, loss of knee movement). 8-Radiographic outcomes: widening of the femoral and tibial reconstruction bone tunnels, deformities, loosening of the screws and others^25, 26, 27, 28, 29^.  If data are available, outcome data for the following time periods will be extracted: short-term follow-up (up to six weeks following treatment), intermediate follow-up (more than six weeks and up to six months after the end of treatment) and long-term follow-up (greater than six months after the end of the intervention). | |
| **METHODS** |  |  | |
| Eligibility criteria | 8 | Specify the study characteristics (such as PICO, study design, setting, time frame) and report characteristics (such as years considered, language, publication status) to be used as criteria for eligibility for the review ObjectivesThis review aims to analyse the effectiveness of the interventions (surgical and conservative) for treating PCL injuries of the knee in adults.MethodsStudy guidelines and registration The authors will perform this systematic review and meta-analysis following the Cochrane Handbook for Systematic Reviews of Interventions, and they will report it in compliance with the Preferred Reporting Items for Systematic Reviews and Meta-Analyses (PRISMA) statement^21, 22, 23, 24^. This review will begin on 2 April 2024, and the expected date to finish is December 2025.  A predetermined written protocol is registered on the PROSPERO platform under number CRD42023430493. Patient and public involvement This protocol is for a systematic review and meta-analysis; therefore, it will only analyse randomized clinical trials. As there is no patient involvement, there is no need for informed consent. Eligibility criteria The authors will only include randomized clinical trials, studies performed in humans, and trials comparing any interventions for treating PCL injuries of the knee in adults. There will be no restrictions on the language or the year of publication. Population The authors will include trials involving adults over 17 years of age who were diagnosed with posterior cruciate ligament injuries (unilateral or bilateral) of the knee confirmed by magnetic resonance (MR) imaging.  **Intervention**  Randomized clinical trials comparing any methods of operative and conservative (nonsurgical treatment) interventions and comparisons with each other for the treatment of PCL injuries in adults.  **Comparator**  Any surgical or non-surgical treatment used for treating PCL injuries or no treatment.  **Outcomes** *Primary outcomes:* 1-Validated health-related quality of life scores (36-item Short Form (SF-36); (Ware) or others). 2-Validated patient-rated measures of knee function (the Lysholm Knee Scale, Cincinnati Knee Rating Scale or others). 3- Pain^25, 26, 27, 28, 29^.  *Secondary outcomes:* 1- Observer-rated measures of knee function (Roos; Tegner, and others). 2- Knee strength and range of motion. 3- Patient satisfaction with treatment. 4-Infection. 5-Treatment failure. 6-Time of rehabilitation and time to return to previous activities (labour, sports and others). 7-Adverse outcomes: early complications (wound infection or dehiscence, arthrofibrosis, paraesthesia, pain, loss of knee movement, and others); short-term complications (skin problems, stiffness, hardware irritation and loosening, loss of knee movement); and late complications (arthrofibrosis, sensitive or painful wound site, hardware irritation or loosening, loss of knee movement). 8-Radiographic outcomes: widening of the femoral and tibial reconstruction bone tunnels, deformities, loosening of the screws and others^25, 26, 27, 28, 29^.  If data are available, outcome data for the following time periods will be extracted: short-term follow-up (up to six weeks following treatment), intermediate follow-up (more than six weeks and up to six months after the end of treatment) and long-term follow-up (greater than six months after the end of the intervention). Study types This systematic review and meta-analysis will only include randomized controlled trials (RCTs). Other studies will be excluded. Search strategy The authors will search the electronic databases until April 2024 for published literature of RCTs to identify eligible studies. These include the PubMed, Cochrane Library, LILACS and EMBASE databases. Searching other resources The authors will search reference lists from all included articles, reviews and textbooks for possible relevant studies, and they will contact experts in the field. They will search for relevant reviews on the database of [Health Technology Assessment (HTA)](https://database.inahta.org/)  and the [Database of Abstracts of Reviews of Effects (DARE)](https://www.crd.york.ac.uk/crdweb/), and any errata or retraction from the eligible trials on [PubMed](http://www.pubmed.ncbi.nlm.nih.gov/), and they will report the date of the search. The authors will also search conference proceedings of the American Academy of Orthopaedic Surgeons and search for gray literature on [ProQuest Dissertations and Theses](https://about.proquest.com/en/products-services/pqdtglobal/) and [www.opengrey.eu](http://www.opengrey.eu/) ^30, 31^. | |
| Information sources | 9 | Describe all intended information sources (such as electronic databases, contact with study authors, trial registers or other grey literature sources) with planned dates of coverage Search strategy The authors will search the electronic databases until April 2024 for published literature of RCTs to identify eligible studies. These include the PubMed, Cochrane Library, LILACS and EMBASE databases. Searching other resources The authors will search reference lists from all included articles, reviews and textbooks for possible relevant studies, and they will contact experts in the field. They will search for relevant reviews on the database of [Health Technology Assessment (HTA)](https://database.inahta.org/)  and the [Database of Abstracts of Reviews of Effects (DARE)](https://www.crd.york.ac.uk/crdweb/), and any errata or retraction from the eligible trials on [PubMed](http://www.pubmed.ncbi.nlm.nih.gov/), and they will report the date of the search. The authors will also search conference proceedings of the American Academy of Orthopaedic Surgeons and search for gray literature on [ProQuest Dissertations and Theses](https://about.proquest.com/en/products-services/pqdtglobal/) and [www.opengrey.eu](http://www.opengrey.eu/) ^30, 31^. | |
| Search strategy | 10 | Present draft of search strategy to be used for at least one electronic database, including planned limits, such that it could be repeated  **. Eletronic search strategies presented to the main databases**  (MEDLINE/ PUBMED)  Search Strategy: 1 Posterior Cruciate Ligament/  2 ((posterior and cruciate and ligament) or PCL).tw.  3 1 or 2  4 exp Surgical Procedures, Operative/  5 exp Orthopedic Fixation Devices/  6 Orthopedic Procedures/  7 (surg* or operat* or reconstruct* or replace* or repair* or graft*).tw.  8 su.fs.  9 or/4-8  10 exp Physical Therapy Modalities/  11 exp Rehabilitation/  12 exp Immobilization/  13 exp Anti-Inflammatory Agents/  14 Conservative Treatment/  15 Braces/ or Crutches/  16 (non-surg* or nonsurg* or non-operat* or nonoperat* or conserv* or rehab* or physiotherapy or physical therapy or exercis* or brace* or cast* or plaster or crutches or immobli* or anti-inflammatory or non-steriod).tw.  17 or/10-16  18 9 or 17  19 3 and 18  20 randomized controlled trial.pt.  21 controlled clinical trial.pt.  22 randomi?ed.ab.  23 placebo.ab.  24 drug therapy.fs.  25 randomly.ab.  26 trial.ab.  27 groups.ab.  28 or/20-27  29 exp animals/ not humans.sh.  30 28 not 29  31 19 and 30   - **This search strategy will be modified as required for other electronic databases.** | |
| Study records: |  |  | |
| Data management | 11a | Describe the mechanism(s) that will be used to manage records and data throughout the review  **Study Selection**  Two authors will screen abstracts and titles for inclusion of all the potentially relevant trials identified as a result of the search and code them as 'retrieve' (eligible, potentially eligible, or unclear) or not retrieved. The authors will retrieve the full text of the study reports or publications, and two review authors will independently screen the full text to identify trials for inclusion and record reasons for exclusion of the ineligible trials. Disagreements will be resolved by discussion and, if necessary, by a third author.  The review authors will use an Excel spreadsheet to record the decision-making, containing an explanation regarding the inclusion or exclusion of the study. If any trial is incomplete regarding the necessary information, they will send an email to the author for clarification, and if the original author does not respond, they will designate the study as ‘missing information’  **Data extraction**  The analysis will be conducted using Microsoft Excel (Microsoft Corporation, Redmond, Washington, USA). Two review authors will independently perform data extraction using a data collection form piloted on all included studies. The authors will independently extract the following data from each eligible trial:  *Methods*: Study design, study duration, study locations, study setting, date of study and funding of the study.  *Characteristics of the population (participants)*: sample size (N), number randomized, number lost (to follow-up and/or withdrawn), age range, mean age, sex, severity of the condition, diagnostic criteria, inclusion and exclusion criteria, unilateral or bilateral injury, classification of the PCL lesion, partial or complete lesion, and presence or absence of concomitant lesions.  *Intervention*: all details of any intervention (nonsurgical or surgical) and its comparison.  *Outcomes (including measures and time points)*  *Numerical data for outcomes of interest*  *Type of analysis/analyses presented*  *Notes*: notable declarations of interest of trial authors and funding for trials. Details will be obtain if it was possible to contact the authors of the trials.  *Information needed to assess bias* *(e.g., presence or absence of a protocol, a new or old device or technique, deviations from intended interventions, use of data imputed for key outcomes, etc.).*  *Information needed to assess the Grading of Recommendations, Assessment, Development and Evaluation (GRADE) (e.g., baseline risk in the control group for key outcomes, heterogeneity, indirectness, etc.).*  Disagreements will be resolved by a third author.  All the data collected will be recorded in Revman 5.4, the Cochrane Library software. | |

| Selection process | 11b | State the process that will be used for selecting studies (such as two independent reviewers) through each phase of the review (that is, screening, eligibility and inclusion in meta-analysis)  **Study Selection**  Two authors will screen abstracts and titles for inclusion of all the potentially relevant trials identified as a result of the search and code them as 'retrieve' (eligible, potentially eligible, or unclear) or not retrieved. The authors will retrieve the full text of the study reports or publications, and two review authors will independently screen the full text to identify trials for inclusion and record reasons for exclusion of the ineligible trials. Disagreements will be resolved by discussion and, if necessary, by a third author.  The review authors will use an Excel spreadsheet to record the decision-making, containing an explanation regarding the inclusion or exclusion of the study. If any trial is incomplete regarding the necessary information, they will send an email to the author for clarification, and if the original author does not respond, they will designate the study as ‘missing information’ |
| --- | --- | --- |
| Data collection process | 11c | Describe planned method of extracting data from reports (such as piloting forms, done independently, in duplicate), any processes for obtaining and confirming data from investigators  **Data extraction**  The analysis will be conducted using Microsoft Excel (Microsoft Corporation, Redmond, Washington, USA). Two review authors will independently perform data extraction using a data collection form piloted on all included studies. The authors will independently extract the following data from each eligible trial:  *Methods*: Study design, study duration, study locations, study setting, date of study and funding of the study.  *Characteristics of the population (participants)*: sample size (N), number randomized, number lost (to follow-up and/or withdrawn), age range, mean age, sex, severity of the condition, diagnostic criteria, inclusion and exclusion criteria, unilateral or bilateral injury, classification of the PCL lesion, partial or complete lesion, and presence or absence of concomitant lesions.  *Intervention*: all details of any intervention (nonsurgical or surgical) and its comparison.  *Outcomes (including measures and time points)*  *Numerical data for outcomes of interest*  *Type of analysis/analyses presented*  *Notes*: notable declarations of interest of trial authors and funding for trials. Details will be obtain if it was possible to contact the authors of the trials.  *Information needed to assess bias* *(e.g., presence or absence of a protocol, a new or old device or technique, deviations from intended interventions, use of data imputed for key outcomes, etc.).*  *Information needed to assess the Grading of Recommendations, Assessment, Development and Evaluation (GRADE) (e.g., baseline risk in the control group for key outcomes, heterogeneity, indirectness, etc.).*  Disagreements will be resolved by a third author. |
| Data items | 12 | List and define all variables for which data will be sought (such as PICO items, funding sources), any pre-planned data assumptions and simplifications  The authors will independently extract the following data from each eligible trial:  *Methods*: Study design, study duration, study locations, study setting, date of study and funding of the study.  *Characteristics of the population (participants)*: sample size (N), number randomized, number lost (to follow-up and/or withdrawn), age range, mean age, sex, severity of the condition, diagnostic criteria, inclusion and exclusion criteria, unilateral or bilateral injury, classification of the PCL lesion, partial or complete lesion, and presence or absence of concomitant lesions.  *Intervention*: all details of any intervention (nonsurgical or surgical) and its comparison.  *Outcomes (including measures and time points)*  *Numerical data for outcomes of interest*  *Type of analysis/analyses presented*  *Notes*: notable declarations of interest of trial authors and funding for trials. Details will be obtain if it was possible to contact the authors of the trials.  *Information needed to assess bias* *(e.g., presence or absence of a protocol, a new or old device or technique, deviations from intended interventions, use of data imputed for key outcomes, etc.).*  *Information needed to assess the Grading of Recommendations, Assessment, Development and Evaluation (GRADE) (e.g., baseline risk in the control group for key outcomes, heterogeneity, indirectness, etc.).*  Disagreements will be resolved by a third author. |
| Outcomes and prioritization | 13 | List and define all outcomes for which data will be sought, including prioritization of main and additional outcomes, with rationale  **Outcomes** *Primary outcomes:* 1-Validated health-related quality of life scores (36-item Short Form (SF-36); (Ware) or others). 2-Validated patient-rated measures of knee function (the Lysholm Knee Scale, Cincinnati Knee Rating Scale or others). 3- Pain^25, 26, 27, 28, 29^.  *Secondary outcomes:* 1- Observer-rated measures of knee function (Roos; Tegner, and others). 2- Knee strength and range of motion. 3- Patient satisfaction with treatment. 4-Infection. 5-Treatment failure. 6-Time of rehabilitation and time to return to previous activities (labour, sports and others). 7-Adverse outcomes: early complications (wound infection or dehiscence, arthrofibrosis, paraesthesia, pain, loss of knee movement, and others); short-term complications (skin problems, stiffness, hardware irritation and loosening, loss of knee movement); and late complications (arthrofibrosis, sensitive or painful wound site, hardware irritation or loosening, loss of knee movement). 8-Radiographic outcomes: widening of the femoral and tibial reconstruction bone tunnels, deformities, loosening of the screws and others^25, 26, 27, 28, 29^.  If data are available, outcome data for the following time periods will be extracted: short-term follow-up (up to six weeks following treatment), intermediate follow-up (more than six weeks and up to six months after the end of treatment) and long-term follow-up (greater than six months after the end of the intervention). |
| Risk of bias in individual studies | 14 | Describe anticipated methods for assessing risk of bias of individual studies, including whether this will be done at the outcome or study level, or both; state how this information will be used in data synthesis  **Risk of bias assessment**  Two authors will independently assess the risk of bias of the included studies using the Risk of Bias 2 tool (a revised Cochrane risk-of-bias tool for randomized trials). The risk of bias will be assessed for all predefined primary and secondary outcomes^32, 33^. The following five methodological domains will be evaluated for risk of bias: (1) arising from the randomization process, (2) due to deviations from the intended interventions, (3) due to missing outcome data, (4) in measurement of the outcome and (5) in selection of the reported result.  A ‘low’ risk of bias, ‘high’ risk of bias or ‘some concerns’ judgement will be assigned to each domain; the latter reflecting a lack of information or uncertainty about the potential for bias. Any disagreements between the review authors regarding the risk of bias for each domain will be resolved by discussion or by a third author, if necessary^34, 35, 36^. |
| Data synthesis | 15a | Describe criteria under which study data will be quantitatively synthesized  **Statistical analyses**  **Measures of treatment effect**  The authors will express dichotomous data as risk ratios (RRs) with 95% confidence intervals (CIs). For continuous outcomes measured on the same scale (e.g., body weight in kg), the intervention effect will be estimated using the mean difference (MD) with the corresponding standard deviation (SD) and 95% CIs. For continuous outcomes that measure the same underlying concept (e.g., knee functional score, health‐related quality of life) but use different measurement scales, the standardized mean difference (SMD) will be calculated. The magnitude of the SMD will be interpreted according to Cohen (small/minor SMD: 0.2 or less, medium SMD: 0.2 to 0.8, large SMD: 0.8 or greater)^36, 37, 38^.  **Unit of analysis issues:** The unit of randomization for the included studies is likely to be individual participants. When studies included patients with bilateral posterior cruciate ligament lesions, the data may be evaluated for lesions rather than for individual patients. When such a unit of analysis issues arises and appropriate corrections have not been made, the authors will consider presenting data for such trials only where the disparity between the unit of analysis and randomization is small. The authors will not include crossover or cluster-randomized trials in this review. |
|  | 15b | If data are appropriate for quantitative synthesis, describe planned summary measures, methods of handling data and methods of combining data from studies, including any planned exploration of consistency (such as I^2^, Kendall’s τ)  **Statistical analyses**  **Measures of treatment effect**  The authors will express dichotomous data as risk ratios (RRs) with 95% confidence intervals (CIs). For continuous outcomes measured on the same scale (e.g., body weight in kg), the intervention effect will be estimated using the mean difference (MD) with the corresponding standard deviation (SD) and 95% CIs. For continuous outcomes that measure the same underlying concept (e.g., knee functional score, health‐related quality of life) but use different measurement scales, the standardized mean difference (SMD) will be calculated. The magnitude of the SMD will be interpreted according to Cohen (small/minor SMD: 0.2 or less, medium SMD: 0.2 to 0.8, large SMD: 0.8 or greater)^36, 37, 38^.  **Unit of analysis issues:** The unit of randomization for the included studies is likely to be individual participants. When studies included patients with bilateral posterior cruciate ligament lesions, the data may be evaluated for lesions rather than for individual patients. When such a unit of analysis issues arises and appropriate corrections have not been made, the authors will consider presenting data for such trials only where the disparity between the unit of analysis and randomization is small. The authors will not include crossover or cluster-randomized trials in this review. Assessment of heterogeneity The heterogeneity of estimate effects between the included trials will be assessed by visual inspection of the forest plot (analysis) along with consideration of the chi-squared test for heterogeneity and the I-squared statistic. The authors will define heterogeneity based on I^2^ values as follows: 25%, low heterogeneity; 50%, moderate heterogeneity; and 90%, high heterogeneity. All the statistical analyses will be performed using Review Manager V.5.4 software, with p value of <0.05 considered statistically significant. Assessment of reporting biases The review authors will assess publication bias by visually checking funnel plot asymmetry where sufficient trials and data are available^35,36^.  The authors will create a funnel plot to explore possible small study biases if there are at least 10 trials included in the meta‐analysis. The funnel plots will be interpreted to determine the possible reasons for funnel plot asymmetry, as described in Chapter 13 of the *Cochrane Handbook for Systematic Reviews of Interventions.* If the authors are able to pool more than 10 trials, they will undertake formal statistical tests to investigate funnel plot asymmetry and will follow the recommendations in section 13.3 of the *Cochrane Handbook for Systematic Reviews of Interventions^35,36^*.  To assess outcome reporting bias, the trial protocols will be checked against published reports. The authors will evaluate whether selective reporting of outcomes is present, too^35,36^. Subgroup analysis The authors will investigate surgical and nonsurgical management of posterior cruciate ligament injuries.  The following data will be analysed: type of posterior cruciate ligament injury (total or partial, acute or chronic), mechanism of injury (caused by high energy or low energy), age of the patients, type (technique) of the surgery or conservative treatment, whether the PCL lesion or injury was partial or complete, and whether the injury was isolated or concomitant.  The formal test for subgroup intersection will be used in Review Manager 5.4/Rev.Man. The review authors will use Revman web software and interpret the results as advised in section 10.11.2 of the *Cochrane Handbook for Systematic Reviews of Interventions*. The authors will compare the magnitude of the effect between subgroups by assessing the overlap between CIs (interval confidence) of the summary effect estimates, where non‐overlapping CIs indicate statistical significance.  **Data synthesis**  **Strategy for data synthesis**  The results of comparable groups of trials will be pooled when possible and appropriate (if participants, interventions, comparisons and outcomes are sufficiently similar in the included trials). The authors will conduct all meta‐analyses using Review Manager 5.4/Rev.Man. Web software. It is anticipated that the underlying effect of the intervention may vary between studies, as there are likely differences between participants, settings and the interventions used for each study. Therefore, the authors will use a random‐effects method for meta‐analysis^37, 38, 39^.  Estimation effects will be expressed as the number needed to treat (NNT) when appropriate.  For dichotomous data, treatment differences will be analysed as a risk ratio (RR) calculated using the Mantel‐Haenszel method.  For continuous outcomes, if all data was from the same scale, the mean follow‐up values will be pooled with change‐from‐baseline data, and the results will be reported as the mean difference. If there is a need to report standardized mean differences, then the review authors will not pool endpoint and change‐from‐baseline data.  If the included studies do not allow pooling of data, the review authors will present the results in a narrative format^38, 39, 40^. Sensitivity analysis The review authors plan to carry out the following sensitivity analyses for the main comparisons to investigate the robustness of the treatment effect on critical outcomes:  Impact of including studies with high or unclear risk of detection, selection, and attrition biases  Impact of including studies with imputed data  The Grading of Recommendations, Assessment, Development and Evaluation (GRADE) approach will be used to assess the quality of evidence related to each of the primary outcomes listed^41,42^. |
|  | 15c | Describe any proposed additional analyses (such as sensitivity or subgroup analyses, meta-regression) Subgroup analysis The authors will investigate surgical and nonsurgical management of posterior cruciate ligament injuries.  The following data will be analysed: type of posterior cruciate ligament injury (total or partial, acute or chronic), mechanism of injury (caused by high energy or low energy), age of the patients, type (technique) of the surgery or conservative treatment, whether the PCL lesion or injury was partial or complete, and whether the injury was isolated or concomitant.  The formal test for subgroup intersection will be used in Review Manager 5.4/Rev.Man. The review authors will use Revman web software and interpret the results as advised in section 10.11.2 of the *Cochrane Handbook for Systematic Reviews of Interventions*. The authors will compare the magnitude of the effect between subgroups by assessing the overlap between CIs (interval confidence) of the summary effect estimates, where non‐overlapping CIs indicate statistical significance. Sensitivity analysis The review authors plan to carry out the following sensitivity analyses for the main comparisons to investigate the robustness of the treatment effect on critical outcomes:  Impact of including studies with high or unclear risk of detection, selection, and attrition biases  Impact of including studies with imputed data  The Grading of Recommendations, Assessment, Development and Evaluation (GRADE) approach will be used to assess the quality of evidence related to each of the primary outcomes listed^41,42^. |
|  | 15d | If quantitative synthesis is not appropriate, describe the type of summary planned  If the included studies do not allow pooling of data, the review authors will present the results in a narrative format^38^ |
| Meta-bias(es) | 16 | Specify any planned assessment of meta-bias(es) (such as publication bias across studies, selective reporting within studies) |
|  |  | Assessment of reporting biases The review authors will assess publication bias by visually checking funnel plot asymmetry where sufficient trials and data are available^35,36^.  The authors will create a funnel plot to explore possible small study biases if there are at least 10 trials included in the meta‐analysis. The funnel plots will be interpreted to determine the possible reasons for funnel plot asymmetry, as described in Chapter 13 of the *Cochrane Handbook for Systematic Reviews of Interventions.* If the authors are able to pool more than 10 trials, they will undertake formal statistical tests to investigate funnel plot asymmetry and will follow the recommendations in section 13.3 of the *Cochrane Handbook for Systematic Reviews of Interventions^35,36^*.  To assess outcome reporting bias, the trial protocols will be checked against published reports. The authors will evaluate whether selective reporting of outcomes is present, too |
| Confidence in cumulative evidence | 17 | Describe how the strength of the body of evidence will be assessed (such as GRADE) Confidence in cumulative evidence The quality of evidence for each outcome will be assessed using GRADE ^41, 42, 43, 44^. The quality of the studies’ outcomes will be classified into four categories: high, moderate, low or very low. The authors will create “Summary of Finding Table” for the following outcomes: 1-Validated health-related quality of life scores. 2-Validated patient-rated measures of knee function. 3- Pain: ^44, 45, 46, 47^.  The authors will use the methods and recommendations described in section 8.5, chapter 12 of the Cochrane Handbook for Systematic Reviews of Interventions, using GRADE Pro software^32, 35,42,45,47^. |

* It is strongly recommended that this checklist be read in conjunction with the PRISMA-P Explanation and Elaboration (cite when available) for important clarification on the items. Amendments to a review protocol should be tracked and dated. The copyright for PRISMA-P (including checklist) is held by the PRISMA-P Group and is distributed under a Creative Commons Attribution Licence 4.0.

*From: Shamseer L, Moher D, Clarke M, Ghersi D, Liberati A, Petticrew M, Shekelle P, Stewart L, PRISMA-P Group. Preferred reporting items for systematic review and meta-analysis protocols (PRISMA-P) 2015: elaboration and explanation. BMJ. 2015 Jan 2;349(jan02 1):g7647.*
