# Supplementary material for: Interventions for treating posterior cruciate ligament injuries of the knee in adults: A systematic review and meta-analysis protocol
Source: PLoS One. 2025 Sep 25;20(9):e0333015. doi: 10.1371/journal.pone.0333015 (PMC12463263; doi:10.1371/journal.pone.0333015)
Supplement: Attachment — Submitted filename: Response to Reviewers (1).pdf [file pone.0333015.s003.pdf]

Response to Reviewers:

"For the study protocol to be considered for publication it should clearly address the suggestions from reviewer 2, and discuss how the findings are likely to be beneficial beyond what is currently available in the existing literature. "

Reviewer 2:

6. Here are some optional suggestions and comments that might be helpful for the authors in planning and refining their study:

Clarification of Inclusion Criteria: While the inclusion criteria for selecting studies are generally clear, it may be beneficial to provide more detailed justification for specific choices, such as the age range of participants or the types of interventions considered. This can help readers understand the scope and relevance of the review.

Thank you for the suggestions. We agree with them and we corrected the text as follows:

"**Population**

The authors will include trials involving adults over 17 years of age (at this age the bone growth lines are closing or are closed what modify the treatment between children and adults) who were diagnosed with posterior cruciate ligament injuries (unilateral or bilateral) of the knee confirmed by magnetic resonance (MR) imaging.

Intervention

Randomized clinical trials comparing any methods of operative and conservative (nonsurgical treatment) interventions and comparisons with each other for the treatment of PCL injuries in adults. The interventions above were chosen because they contemplate all interventions to treat posterior cruciate ligament injuries of the knee that may exist in the literature."

Addressing Publication Bias: Consider discussing how you plan to assess and address potential publication bias, such as using funnel plots or statistical tests. This would strengthen the credibility of the systematic review's findings.

Thank you for the suggestions and comments. We agree with them. But, we placed this part in the first protocol as follows (highlighted in yellow):

Assessment of reporting biases

The review authors will assess publication bias by visually checking funnel plot asymmetry where sufficient trials and data are available[35,36].

The authors will create a funnel plot to explore possible small study biases if there are at least 10 trials included in the meta-analysis. The funnel plots will be interpreted to determine the possible reasons for funnel plot asymmetry, as described in Chapter 13 of the *Cochrane Handbook for Systematic Reviews of Interventions*. If the authors are able to pool more than 10 trials, they will undertake formal statistical tests to investigate funnel plot asymmetry and will follow the recommendations in section 13.3 of the *Cochrane Handbook for Systematic Reviews of Interventions*[35,36].

Detailed Search Strategy: Although the manuscript outlines the databases to be searched, providing a detailed search strategy, including specific keywords and Boolean operators, could enhance transparency and replicability. This detail would also allow others to understand the comprehensiveness of your search.

Thank you for the suggestions and comments. We agree with them. But, we placed this part in the first protocol as follows (highlighted in yellow):

The electronic search strategy is presented in **Table 1.**

Table 1. Eletronic search strategies presented to the main databases

(MEDLINE/ PUBMED)

Search Strategy: 1 Posterior Cruciate Ligament/

2 ((posterior and cruciate and ligament) or PCL).tw.

3 1 or 2

4 exp Surgical Procedures, Operative/

5 exp Orthopedic Fixation Devices/

6 Orthopedic Procedures/

7 (surg* or operat* or reconstruct* or replace* or repair* or graft*).tw.

8 su.fs.

9 or/4-8

10 exp Physical Therapy Modalities/

11 exp Rehabilitation/

12 exp Immobilization/

13 exp Anti-Inflammatory Agents/

14 Conservative Treatment/

15 Braces/ or Crutches/

16 (non-surg* or nonsurg* or non-operat* or nonoperat* or conserv* or rehab* or physiotherapy or physical therapy or exercis* or brace* or cast* or plaster or crutches or immobli* or anti-inflammatory or non-steriod).tw.

17 or/10-16

18 9 or 17

19 3 and 18

20 randomized controlled trial.pt.

21 controlled clinical trial.pt.

22 randomi?ed.ab.

23 placebo.ab.

24 drug therapy.fs.

25 randomly.ab.

26 trial.ab.

27 groups.ab.

28 or/20-27

29 exp animals/ not humans.sh.

30 28 not 29

Following your suggestions, we provide a detailed search strategy (including keywords and Boolean operators for Embase, Cochrane Library and LILACS:

Table 2: Eletronic search strategies presented to the main databases

(Cochrane library)

[mh ^"Posterior Cruciate Ligament"]
((posterior:ti,ab AND cruciate:ti,ab AND ligament:ti,ab) OR PCL:ti,ab)
#1 OR #2
[mh "Surgical Procedures, Operative"]
[mh "Orthopedic Fixation Devices"]
[mh ^"Orthopedic Procedures"]
(surg*:ti,ab OR operat*:ti,ab OR reconstruct*:ti,ab OR replace*:ti,ab OR repair*:ti,ab OR graft*:ti,ab)
[mh /SU]
#4 OR #5 OR #6 OR #7 OR #8
[mh "Physical Therapy Modalities"]
[mh Rehabilitation]
[mh Immobilization]
[mh "Anti-Inflammatory Agents"]
[mh ^"Conservative Treatment"]
[mh ^Braces] OR [mh ^Crutches]
(non-surg*:ti,ab OR nonsurg*:ti,ab OR non-operat*:ti,ab OR nonoperat*:ti,ab OR conserv*:ti,ab OR rehab*:ti,ab OR physiotherapy:ti,ab OR "physical therapy":ti,ab OR exercis*:ti,ab OR brace*:ti,ab OR cast*:ti,ab OR plaster:ti,ab OR crutches:ti,ab OR immobli*:ti,ab OR anti-inflammatory:ti,ab OR non-steriod:ti,ab)
#10 OR #11 OR #12 OR #13 OR #14 OR #15 OR #16
#9 OR #17
#3 AND #18
"randomized controlled trial":pt
"controlled clinical trial":pt
randomi?ed:ab
placebo:ab
[mh /DT]
randomly:ab
trial:ab
groups:ab
#20 OR #21 OR #22 OR #23 OR #24 OR #25 OR #26 OR #27
[mh animals] NOT [mh ^humans]
#28 NOT #29

(EMBASE)

"Posterior Cruciate Ligament"/
((posterior AND cruciate AND ligament) OR PCL).tw.
1 OR 2
exp "Surgical Procedures, Operative"/
exp "Orthopedic Fixation Devices"/
"Orthopedic Procedures"/
(surg* OR operat* OR reconstruct* OR replace* OR repair* OR graft*).tw.
"Surgery".fs.
4 OR 5 OR 6 OR 7 OR 8
exp "Physical Therapy Modalities"/
exp Rehabilitation/
exp Immobilization/
exp "Anti-Inflammatory Agents"/
"Conservative Treatment"/
Braces/ OR Crutches/
(non-surg* OR nonsurg* OR non-operat* OR nonoperat* OR conserv* OR rehab* OR physiotherapy OR "physical therapy" OR exercis* OR brace* OR cast* OR plaster OR crutches OR immobli* OR anti-inflammatory OR non-steriod).tw.
10 OR 11 OR 12 OR 13 OR 14 OR 15 OR 16
9 OR 17
3 AND 18
"randomized controlled trial".pt.
"controlled clinical trial".pt.
randomi?ed.ab.
placebo.ab.
"Drug Therapy".fs.
randomly.ab.
trial.ab.
groups.ab.
20 OR 21 OR 22 OR 23 OR 24 OR 25 OR 26 OR 27
exp animals/ NOT humans/
28 NOT 29

Table 3. Eletronic search strategies presented to the main databases

(LILACS)

1 ("ligamento cruzado posterior")

2 (ligamento AND cruzado AND posterior) OR PCL

3 1 OR 2

4 ("procedimientos quirúrgicos")

5 ("fijacion ortopédica")

6 ("procedimientos ortopédicos")

7 (cirúrg* OR operador* OR reconstr* OR substitu* OR repar* OR enxert*)

8 ("procedimientos quirúrgicos"[DeCS])

9 4 OR 5 OR 6 OR 7 OR 8

10 ("fisioterapia")

11 ("rehabilitación")

12 ("inmovilización")

13 ("agentes antiinflamatorios")

14 ("tratamento conservador")

15 (brac* OR muleta*)

16 (conservative OR nonsurgical OR nonoperativa OR physiotherapy OR physical OR exerc* OR brace OR plaster OR crutches OR immobi* OR non-steroidal OR rehab*)

17 10 OR 11 OR 12 OR 13 OR 14 OR 15 OR 16

18 9 OR 17

19 3 AND 18

20 (ensayo clínico controlado OR ensayo controlado)

21 (aleatorizado OR randomized)

22 (placebo)

23 (terapia)

24 (drug OR fármaco)

25 (ensayo OR trial)

26 (grupos OR groups)

27 20 OR 21 OR 22 OR 23 OR 24 OR 25 OR 26

28 (animal OR animales) NOT (humano OR humanos)

29 27 NOT 28

Additional Sensitivity Analyses: Including additional sensitivity analyses, such as excluding studies with a high risk of bias or those with small sample sizes, could provide more nuanced insights into the robustness of your findings.

e.g

Cernat E M, Neagu A, Betianu C, Manolescu L, Avram G, Pogarasteanu M-E, Barbilian A. 2024. Balancing Anterior and Posterior Cruciate Ligaments in Adults. Cureus 16(5): e59683. DOI: 10.7759/cureus.59683

Cernat, E.M.; Dima, A.; Popescu, C.; Neagu, A.; Betianu, C.; Moga, M.; Manolescu, L.; Barbilian, A. Anterior Intercondylar Notch Geometry in Relation to the Native Anterior Cruciate Ligament Size. J. Clin. Med. 2024, 13, 309. https://doi.org/10.3390/jcm13020309

Thank you for the suggestions and comments. We agree with them. But, we placed this part in the first protocol as follows (highlighted in yellow) and now we improved the text following your suggestions (in green):

Sensitivity analysis

The review authors plan to carry out the following sensitivity analyses for the main comparisons to investigate the robustness of the treatment effect on critical outcomes:

Impact of including studies with high or unclear risk of detection, selection, and attrition biases

Impact of including studies with imputed data.

Impact of excluding trials with a high risk of bias or those with small sample sizes.

The Grading of Recommendations, Assessment, Development and Evaluation (GRADE) approach will be used to assess the quality of evidence related to each of the primary outcomes listed[41,42].

Consideration of Grey Literature: Including grey literature, such as conference abstracts, theses, and technical reports, could help mitigate publication bias and provide a more comprehensive view of the available evidence.

Thank you for the suggestions and comments. We agree with them. But, we placed this part in the first protocol as follows (highlighted in yellow) and, now we improved the text following your suggestions (in green):

Methods and analysis Starting in August 2025, the authors will accomplisha detailed search using the MEDLINE/PubMed, EMBASE, Cochrane Central Register of Controlled Trials and LILACS databases. Relevant gray literature(academic papers, reference lists theses, technical reports and conference abstracts) will also be included.

Two authors will independently screen and extract the information found from randomized controlled trials in the literature. The bias and quality of the included studies will be evaluated using the Risk of Bias 2 tool provided by the Cochrane Collaboration. Statistical analyses will be performed using Review Manager V.5.4/Review Manager Web software.

Searching other resources

The authors will search reference lists from all included articles, reviews and textbooks for possible relevant studies, and they will contact experts in the field. They will search for relevant reviews on the database of Health Technology Assessment (HTA) and the Database of Abstracts of Reviews of Effects (DARE), and any errata or retraction from the eligible trials on PubMed, and they will report the date of the search. The authors will also search conference proceedings of the American Academy of Orthopaedic Surgeons and search for gray literature (such as conference abstracts, theses and technical reports) on ProQuest Dissertations and Theses and www.opengrey.eu [30, 31].

Patient and Public Involvement (PPI): If not already considered, the inclusion of patient and public involvement in the planning stages or interpretation of results could provide valuable insights and enhance the study's relevance to patient care.

Thank you for the suggestions. We agree with them, and we corrected the text as follows:

"Patient and Public Involvemnet (PPI)

This protocol is for a systematic review and meta-analysis; therefore, it will only analyse randomized clinical trials..There is no need for informed consent because there is no patient involvement [20].

The target of this systematic review is to ensure that this study is relevant and useful for both patients and healthcare professionals involved in the treatment of posterior cruciate ligament injuries. The authors plan to invite patients with posterior cruciate ligament injuries of the knee, and healthcare professionals with extensive experience in the treatment of posterior cruciate ligament lesions to only provide feedback on the introduction, review findings and conclusions [48.]

The consumer involvement (Patient and Public) will be important in ensuring that the review is accessible, clear and accurately reports the most relevant information for both patients and healthcare professionals [48]."

The corrections are in green in the protocol:

Patient and public involvement

This protocol is for a systematic review and meta-analysis; therefore, it will only analyse randomized- clinical trials. As there is no patient involvement, there is no need for informed consent. There is no need for informed consent because there is no patient involvement [20].

The target of this systematic review is to ensure that this study is relevant and useful for both patients and healthcare professionals involved in the treatment of posterior cruciate ligament injuries. The authors plan to invite patients with posterior cruciate ligament injuries of the knee, and healthcare professionals with extensive experience in the treatment of posterior cruciate ligament lesions to only provide feedback on the introduction, review findings and conclusions [48].

The consumer involvement (Patient and Public) will be important in ensuring that the review is accessible, clear and accurately reports the most relevant information for both patients and healthcare professionals [48].

Data Management Plan: While the manuscript states that all relevant data will be made available, providing a more detailed data management plan, including where and how the data will be stored and accessed, could clarify this aspect for potential users.

Thank you for the suggestions and comments. We agree with them and we corrected the text as follows:

" Data management

All the extracted data will be stored and accessed in Rayyan software and in Review Manager V.5.4/Review Manager Web software [37]."

Data management

All the extracted data will be stored and accessed in Rayyan software and in Review Manager V.5.4/Review Manager Web software [37].

Future Research Directions: In the discussion section, it might be beneficial to outline potential areas for future research based on anticipated findings. This could help in identifying gaps that need to be addressed in subsequent studies.

Thank you for the suggestions and comments. We agree with them and we corrected the text as follows:

"Future Research Directions

The authors will carry out a Future Research Directions after analyzing the results found in this review. It will identify gaps in the literature that need to be addressed in subsequent studies. Therefore, the authors can point out the flaws and gaps of the existing literature and indicate the best study designs that can be carried out in the future."

Future Research Directions

The authors will carry out a Future Research Directions after analyzing the results found in this review. It will identify gaps in the literature that need to be addressed in subsequent studies. Therefore, the authors can point out the flaws and gaps of the existing literature and indicate the best study designs that can be carried out in the future.

Reviewer 1:

"Reviewer #1: I would like to thank the authors for their effort writing this search strategy. It is well written but I believe it does not reach the stage of publication as no results is there and until then it adds no value to the reader." (only pointed out this single flaw in the review)

We do not agree and below we will explain the reasons and "discuss how the findings are likely to be beneficial beyond what is currently available in the existing literature."

In March 16, 2024 we sent to Plos One, a letter called "An explanation of why your manuscript should be published"

There we found 9 randomised trials that would be included in our study.

Today, searching the literature (in the main databases), we found at least 15 randomised contolled trials that will be included:

1-External fixator for treatment of the sub-acute and chronic multi-ligament-injured knee.Fabio Janson Angelini1 · Camilo Partezani Helito1 · Marcelo Batista Bonadio1 ·Tales Molica Guimarães1 · Ronald Bispo Barreto1 · José Ricardo Pécora1 ·Gilberto Luis Camanho1 · Roberto Freire da Mota e Albuquerque1. Knee Surg Sports Traumatol Arthrosc (2015) 23:3012–3018.DOI 10.1007/s00167-015-3719-0

2- Arthroscopic single- versus double-bundle posterior cruciate ligament reconstructionsusing hamstring autograft. Ching-Jen Wang [a], Lin-Hsiu Weng [a], Chia-Chen Hsu [a], Yi-Sheng Chan [a] [b]Injury. Volume 35, Issue 12, December 2004, Pages 1293-1299

3- Comparison of Single-Bundle and Double-Bundle Isolated Posterior Cruciate Ligament Reconstruction With Allograft: A Prospective, Randomized Study. Yongqian Li, M.D., Jia Li, M.D., Jianzhao Wang, M.D., Shijun Gao, M.D., and Yingze Zhang, M.D. http://dx.doi.org/10.1016/j.arthro.2014.02.035

4- Prospective Randomized Comparison of Knee Stability and Proprioception for Posterior Cruciate Ligament Reconstruction With Autograft, Hybrid Graft, and g-Irradiated Allograft Jia Li, M.D., Fanlong Kong, M.D., Xianda Gao, M.D., Yong Shen, M.D., and Shijun Gao, M.D. http://dx.doi.org/10.1016/j.arthro.2016.04.024

5- Two-stage ligament reconstruction with remnant preservation as treatment of knee dislocation.Wenpu Ma1†, Yiqun Yang1† and Xin Ha2*. Ma *et al. BMC Surgery* (2023) 23:371 .https://doi.org/10.1186/s12893-023-02271-5

6- **Study:** ABSTRACTS PRESENTED AT THE 7TH BIENNIAL CONGRESS OF THE INTERNATIONAL SOCIETY OF ARTHROSCOPY, KNEE SURGERY, AND ORTHOPAEDIC SPORTS MEDICINE| VOLUME 28, ISSUE 9, SUPPLEMENT , E413-E414, SEPTEMBER 2012.

7- Clinical study on reconstruction of posterior cruciate ligament with platelet rich plasma combined with 3-strand peroneus longus tendons WU Shichun, LIN Wenxiang, XU Weihua, LI Honghan Department of Orthopaedics, Zhangzhou Affiliated Hospital of Fujian Medical University, Zhangzhou Fujian, 363000, P.R.China Corresponding author: LIN Wenxiang, Email: lwxgk3192@126.com

8- **Comparison of early effectiveness of arthroscopic suture bridge technique and conventional double tunnel suture technique in treatment of avulsion fracture of posterior cruciate ligament insertion.** WU Shichun, XU Weihua, LIN Wenxiang, LI Honghan *Department of Orthopedics, Zhangzhou Affiliated Hospital of Fujian Medical University, Zhangzhou Fujian, 363000, P.R.China. Corresponding author: LIN Wenxiang, Email: lwxgk3192@126.com*

9- Hinged External Fixation in the Treatment of Knee Dislocations A Prospective Randomized Study. James P. Stannard, MD, Clayton W. Nuelle, MD, Gerald McGwin, PhD, and David A. Volgas, MD. Investigation performed at the Department of Orthopaedic Surgery, University of Missouri, Columbia, Missouri, and the University of Alabama at Birmingham, Birmingham,

Alabama.**COPYRIGHT _ 2014 BY THE JOURNAL OF BONE AND JOINT SURGERY, INCORPORATED**

10- Arthroscopic reduction and internal fixation (ARIF) versus open reduction internal fixation (ORIF) to elucidate the difference for tibial side PCL avulsion fixation: a randomized controlled trial (RCT). Silvampatti Ramaswamy Sundararajan1 · Joseph Babu Joseph1 · Rajagopalakrishnan Ramakanth1 · Amit Kumar Jha1 · Shanmuganathan Rajasekaran2

11- Comparison of autogenous and allogenous posterior cruciate ligament reconstructions of the knee Ching-Jen Wanga,*, Yi-Sheng Chanb, Lin-Hsiu Wenga, Li-Jen Yuanb, Han-Shiang Chenc aDepartment of Orthopedic Surgery, Chang Gung Memorial Hospital at Kaohsiung, 123 Ta-Pei Road, Niao-Sung Hsiang, Kaohsiung 833, Taiwan bDepartment of Orthopedics and Sports Medicine, Chang Gung Memorial Hospital at Lin-Kou, No. 5, Fu Hsing Street, Kueishan, Taoyuan, Taiwan ROC cDepartment of Surgery, Chang Gung Memorial Hospital at Kaohsiung, 123 Ta-Pei Road, Niao-Sung Hsiang, Kaohsiung 833, Taiwan

12- **Functional outcomes of arthroscopic posterior cruciate ligament reconstruction: comparison of anteromedial and anterolateral trans-tibia approach. To Wong · Ching-Jen Wang · Lin-Hsiu Weng · Shan-Ling Hsu · Wen-Yi Chou · June-Ming Chen ·Yi-Sheng Chan**

13- Posterior Cruciate Ligament Reconstructions Preserving Remnant Fibers A Prospective Randomized Study Comparing Arthroscopic Single-Bundle and Double-Bundle. The online version of this article can be found at: DOI: 10.1177/0363546510382206

Am J Sports Med 2011 39: 474 originally published online November 23, 2010 Kyoung Ho Yoon, Dae Kyung Bae, Sang Jun Song, Hyung Jun Cho and Jung Hwan Lee

14- Does Cast Immobilization Contribute to Posterior Stability After Posterior Cruciate Ligament Reconstruction? Kyoung Ho Yoon, M.D., Sung Woo Park, M.D., Sang Hak Lee, M.D., Man Ho Kim, M.D., Soo Yeon Park, Ph.D., and Hoon Oh, M.D. Arthroscopy: The Journal of Arthroscopic and Related Surgery, Vol 29, No 3 (March), 2013: pp 500-506

15- **Single-bundle posterior cruciate ligament reconstruction with remnant preservation: lateral versus medial-sided augmentation technique.** Jin-zhong Zhao MD, Xiao-qiao Huang-Fu MD, Yao-hua He MD, Xing-guang Yang MD Department of Arthroscopic Surgery, Shanghai Sixth People's Hospital, Shanghai Jiaotong University, Shanghai, China.

***Orthopaedic Surgery* (2009), Volume 1, No. 1, 66–73**

Interventions for treating posterior cruciate ligament injuries of the knee in adults. It is an interesting topical. The tear of the PCL is reported to represent 0.65 to 3 % of the total sports related knee injuries . Occurs more in young male adults.

The PCL tears represent 20% of all knee ligament injuries. The PCL is considered the main ligament of the knee. In the last years, greater participation in sporting and recreational activities by the general population, in all around the world, continues to expose more individuals to the risk of posterior cruciate ligament lesion.

PCL injuries can be treated conservatively or surgically. There are many surgical and conservative techniques for treating the PCL injuries, but there is no answer for the best method or technique.

In the literature, there is no systematic review that included only randomized controlled trials in this topic. So, this review is needed.

There is a protocol in this topic, from Cochrane Library: ***Peccin MS, Almeida GJ, Amaro JT, Cohen M, Soares BG, Atallah AN. WITHDRAWN: Interventions for treating posterior cruciate ligament injuries of the knee in adults. Cochrane Database Syst Rev. 2012 Mar 14;2012(3):CD002939. doi: 10.1002/14651858.CD002939.pub3. PMID: 22419285; PMCID: PMC10687500.***

But, this was withdrawn because it did not find any randomised trial in 2012.

doi: 10.1002/14651858.CD002939.pub3.

WITHDRAWN: Interventions for treating posterior cruciate ligament injuries of the knee in adults

Maria Stella Peccin [1], Gustavo Jm Almeida, Joicemar T Amaro, Moisés Cohen, Bernardo Go Soares, Alvaro N Atallah

Affiliations + expand

PMID: 22419285 PMCID: PMC10687500 DOI: 10.1002/14651858.CD002939.pub3

In Cochrane Library, there is no systematic review in this topic.

We found only a systematic review published in 2020 that compared two types of allograft to reconstruct of posterior cruciate ligament injury. It included only two randomised controlled Trials (Comparative Effectiveness and Safety of Allografts and Autografts in Posterior Cruciate Ligament Reconstruction Surgery: A Systematic Review Cecilia de Villiers, M.Sc., B.Sc., Gregor Goetz, M.S.Sc., M.P.H., Patrick Sadoghi, M.D., Ph.D., M.B.A., and Sabine Geiger-Gritsch, D.M.Sc., M.H.Sc., M.Pharm. Arthroscopy, Sports Medicine, and Rehabilitation, Vol 2, No 6 (December), 2020: pp e893-e907).

These two randomised trials will be included in our review.

Our goal is to perform a systematic review (with only randomised controlled trials) level 1 of evidence. Therefore, we want to report the effectiveness of the interventions (surgical and conservative) for treating posterior cruciate ligament injuries of the knee in adults, and if possible show the best method, intervention or technique.

Because of all this, this work is so important and necessary.
