# Supplementary material for: Interventions for treating posterior cruciate ligament injuries of the knee in adults: A systematic review and meta-analysis protocol
Source: PLoS One. 2025 Sep 25;20(9):e0333015. doi: 10.1371/journal.pone.0333015 (PMC12463263; doi:10.1371/journal.pone.0333015)
Supplement: Attachment — Submitted filename: 2 Response to Reviewers.docx [file pone.0333015.s004.docx]

**Response to Reviewers**

6. Review Comments to the Author

Please use the space provided to explain your answers to the questions above and, if applicable, provide comments about issues authors must address before this protocol can be accepted for publication. You may also include additional comments for the author, including concerns about research or publication ethics.

You may also provide optional suggestions and comments to authors that they might find helpful in planning their study.


(Please upload your review as an attachment if it exceeds 20,000 characters)

Reviewer #3: I would like to congratulate the authors for the thorough revisions performed in response to the previous round of comments. All the requested modifications were adequately addressed, and the manuscript is now much clearer and very well written. The study is of high relevance, and I believe it will be of interest to the readership of PLOS ONE.

**Thank you for your considerations and compliments.**

Only a few minor points remain to be clarified before acceptance:

Introduction – Introduction – Although well contextualized, the introduction is somewhat extensive. Consider condensing it and improving the flow by creating stronger connections between paragraphs, as they are currently too short.

**Thank you for the suggestions. We agree with them and we corrected the text .**

Intervention and control – The distinction between what is considered intervention and control remains slightly unclear. Please provide a more explicit description in the Methods section.

**Thank you for the suggestions and comments. We agree and corrected them.**

Study management software – Kindly specify whether any software was used to manage the study selection process, particularly for duplicate removal and screening. This will strengthen the transparency and reproducibility of the review. **Thank you for the suggestions and comments. We agree with them. We will use the Rayyan software as follows:**

Reviewer #4: This protocol has a clear structure and detailed content, but there is still room for improvement, as specified below:

1. The depth of background information is insufficient. Although it mentions that "conservative treatment and surgical treatment each have advantages and disadvantages", it fails to briefly explain the existing research consensus on the "differences in applicable populations" and "preliminary trends in short-term and long-term efficacy" between the two types of treatment. This makes it difficult to highlight the necessity of the study. It is recommended to supplement the content to further emphasize the value of conducting this systematic review.

**Thank you for the suggestions and comments. We agree with them. Wecorrected them in the text.**

2. It is suggested to revise "a systematic review protocol" to "a systematic review and meta-analysis protocol", which can more accurately reflect the study design.

**Thank you for the suggestions and comments. We agree with them. And now our title changed to: “Interventions for treating posterior cruciate ligament injuries of the knee in adults: a systematic review and meta-analysis protocol.”**

3. For "over 17 years of age" → it is recommended to clearly state "≥18 years" to avoid ambiguity.

**Thank you for the suggestions and comments. We agree and corrected them as follows:.**

4. In the Outcomes section, since Pain is listed as a primary outcome, it is advisable to specify the measurement tool (e.g., Visual Analog Scale (VAS) score).

**Thank you for the suggestions and comments. We corrected them as follows:**

5. In the Search Strategy section, the search period ends in April 2024, while the study is scheduled to start in August 2025. Please provide an explanation for this discrepancy. It is recommended to extend the search end date to the present.

**Thank you for the suggestions and comments. We agree with them. We made a mistake, we did not update the search date in the text. Remembering that this protocol was submitted for the first time in March 2024. Now, we corrected it.**

6. Please check the entire text for spelling and grammatical errors. For example, "Strengths and Limitations os this Study" should be corrected to "Strengths and Limitations of this Study".

**Thank you for the suggestions and comments. We agree and corrected them.**
