# Supplementary material for: Interventions for treating posterior cruciate ligament injuries of the knee in adults: A systematic review and meta-analysis protocol
Source: PLoS One. 2025 Sep 25;20(9):e0333015. doi: 10.1371/journal.pone.0333015 (PMC12463263; doi:10.1371/journal.pone.0333015)
Supplement: Peer review history

# Peer review: Decision Letter 0

**DOI:** 10.1371/journal.pone.0333015.r001

**Authors:** Jennifer Tucker

12 Jun 2025

Dear Dr. Sayum Filho,

Thank you for submitting your manuscript to PLOS ONE. After careful consideration, we feel that it has merit but does not fully meet PLOS ONE’s publication criteria as it currently stands. Therefore, we invite you to submit a revised version of the manuscript that addresses the points raised during the review process.

Please submit your revised manuscript by Jul 21 2025 11:59PM. If you will need more time than this to complete your revisions, please reply to this message or contact the journal office at plosone@plos.org . When you're ready to submit your revision, log on to https://www.editorialmanager.com/pone/ and select the 'Submissions Needing Revision' folder to locate your manuscript file.

- A rebuttal letter that responds to each point raised by the academic editor and reviewer(s). You should upload this letter as a separate file labeled 'Response to Reviewers'.
- A marked-up copy of your manuscript that highlights changes made to the original version. You should upload this as a separate file labeled 'Revised Manuscript with Track Changes'.
- An unmarked version of your revised paper without tracked changes. You should upload this as a separate file labeled 'Manuscript'.

If applicable, we recommend that you deposit your laboratory protocols in protocols.io to enhance the reproducibility of your results. Protocols.io assigns your protocol its own identifier (DOI) so that it can be cited independently in the future. For instructions see: https://journals.plos.org/plosone/s/submission-guidelines#loc-laboratory-protocols . Additionally, PLOS ONE offers an option for publishing peer-reviewed Lab Protocol articles, which describe protocols hosted on protocols.io. Read more information on sharing protocols at https://plos.org/protocols?utm_medium=editorial-email&utm_source=authorletters&utm_campaign=protocols .

We look forward to receiving your revised manuscript.

Kind regards,

Jennifer Tucker, PhD

Staff Editor

PLOS ONE

Journal Requirements:

When submitting your revision, we need you to address these additional requirements.

1. Please ensure that your manuscript meets PLOS ONE's style requirements, including those for file naming. The PLOS ONE style templates can be found at https://journals.plos.org/plosone/s/file?id=wjVg/PLOSOne_formatting_sample_main_body.pdf and https://journals.plos.org/plosone/s/file?id=ba62/PLOSOne_formatting_sample_title_authors_affiliations.pdf

2. We noticed you have some minor occurrence of overlapping text with the following previous publication(s), which needs to be addressed:

- https://bmjopen.bmj.com/content/12/11/e062114.full?

In your revision ensure you cite all your sources (including your own works), and quote or rephrase any duplicated text outside the methods section. Further consideration is dependent on these concerns being addressed.

3. Please provide a complete Data Availability Statement in the submission form, ensuring you include all necessary access information or a reason for why you are unable to make your data freely accessible. If your research concerns only data provided within your submission, please write "All data are in the manuscript and/or supporting information files" as your Data Availability Statement.

4. Please update your submission to use the PLOS LaTeX template. The template and more information on our requirements for LaTeX submissions can be found at http://journals.plos.org/plosone/s/latex.

5. Please amend the manuscript submission data (via Edit Submission) to include author Rogerio Teixeira de Carvalh,; Álvaro N Atallah , Fabio T Matsunag, Flávio Faloppa, João Carlos Belloti.

6. Please amend your list of authors on the manuscript to ensure that each author is linked to an affiliation. Authors’ affiliations should reflect the institution where the work was done (if authors moved subsequently, you can also list the new affiliation stating “current affiliation:….” as necessary).

7. Please remove all personal information, ensure that the data shared are in accordance with participant consent, and re-upload a fully anonymized data set.

Note: spreadsheet columns with personal information must be removed and not hidden as all hidden columns will appear in the published file.

Additional guidance on preparing raw data for publication can be found in our Data Policy (https://journals.plos.org/plosone/s/data-availability#loc-human-research-participant-data-and-other-sensitive-data) and in the following article: http://www.bmj.com/content/340/bmj.c181.long.

8. Please review your reference list to ensure that it is complete and correct. If you have cited papers that have been retracted, please include the rationale for doing so in the manuscript text, or remove these references and replace them with relevant current references. Any changes to the reference list should be mentioned in the rebuttal letter that accompanies your revised manuscript. If you need to cite a retracted article, indicate the article’s retracted status in the References list and also include a citation and full reference for the retraction notice.

[Note: HTML markup is below. Please do not edit.]

Reviewers' comments:

Reviewer's Responses to Questions

**Comments to the Author**

1. Does the manuscript provide a valid rationale for the proposed study, with clearly identified and justified research questions?

Reviewer #1: No

Reviewer #2: Yes

**********

2. Is the protocol technically sound and planned in a manner that will lead to a meaningful outcome and allow testing the stated hypotheses?

Reviewer #1: Yes

Reviewer #2: Yes

**********

3. Is the methodology feasible and described in sufficient detail to allow the work to be replicable?

Reviewer #1: Yes

Reviewer #2: Yes

**********

4. Have the authors described where all data underlying the findings will be made available when the study is complete?

The PLOS Data policy

Reviewer #1: Yes

Reviewer #2: Yes

**********

5. Is the manuscript presented in an intelligible fashion and written in standard English?

Reviewer #1: Yes

Reviewer #2: Yes

**********

Please use the space provided to explain your answers to the questions above and, if applicable, provide comments about issues authors must address before this protocol can be accepted for publication. You may also include additional comments for the author, including concerns about research or publication ethics.

You may also provide optional suggestions and comments to authors that they might find helpful in planning their study.

Reviewer #1: I would like to thank the authors for their effort writing this search strategy. It is well written but I believe it does not reach the stage of publication as no results is there and until then it adds no value to the reader.

Reviewer #2: 1.

The manuscript provides a valid rationale for the proposed study by addressing a significant academic problem related to posterior cruciate ligament (PCL) injuries. The study protocol aims to systematically review and analyze various interventions (both surgical and conservative) for treating these injuries in adults. It outlines the importance of this review in assisting orthopedic surgeons and patients in making informed decisions, emphasizing the relevance of the topic due to the prevalence of such injuries.

The research question is well-justified and intends to contribute to the field by offering a comprehensive assessment of the current evidence regarding the effectiveness of different treatment approaches. The authors clearly state the expected benefits, such as improved decision-making and identification of potential areas for clinical and research improvements. The study is also noted for its adherence to systematic review guidelines, such as PRISMA, and the use of robust methodologies like GRADE for assessing the quality of evidence.

Thus, the manuscript establishes a solid foundation for the research, with clearly identified and justified research questions that aim to fill an important gap in the existing literature.

2.

The protocol for the systematic review on interventions for treating posterior cruciate ligament (PCL) injuries in adults appears technically sound and well-structured to achieve meaningful outcomes. The methodology includes a detailed plan for data extraction, risk of bias assessment, and statistical analysis:

Data Extraction and Bias Assessment: The protocol specifies that two authors will independently extract data and assess the risk of bias using the Cochrane Risk of Bias 2 tool. This dual review process, along with adjudication by a third author in case of disagreements, ensures thorough and unbiased data handling.

Handling Missing Data and Descriptive Analysis: The authors plan to perform intention-to-treat analyses, consult trial authors for missing data, and use best-case and worst-case scenarios to handle data loss. This comprehensive approach helps mitigate the impact of incomplete data on the review's conclusions.

Statistical and Sensitivity Analyses: The statistical methods include the use of risk ratios (RRs) and mean differences (MDs) for dichotomous and continuous outcomes, respectively. The authors also plan to assess heterogeneity and conduct subgroup analyses to explore variations in intervention effects across different patient and treatment characteristics. This methodical approach is critical for identifying factors that may influence the effectiveness of the interventions.

Outcome Measures: The study aims to analyze various outcomes, including the effectiveness of surgical versus conservative treatments. The use of standardized mean differences and consideration of both endpoint and change-from-baseline data further solidifies the analytical framework

3.

The methodology described in the manuscript is feasible and sufficiently detailed to enable replication.

4.

The manuscript indicates that all relevant data from the study will be made available upon completion. This commitment ensures transparency and accessibility, aligning with standard practices for data availability in research

5. The manuscript is presented in an intelligible fashion and written in standard English. The language used throughout is clear and appropriate for a scientific audience. The manuscript is well-structured, following a logical flow that includes the introduction, methods, results, and discussion sections. Each section is detailed and uses proper technical terminology, making the content accessible and understandable to readers familiar with the subject matter

6. Here are some optional suggestions and comments that might be helpful for the authors in planning and refining their study:

Clarification of Inclusion Criteria: While the inclusion criteria for selecting studies are generally clear, it may be beneficial to provide more detailed justification for specific choices, such as the age range of participants or the types of interventions considered. This can help readers understand the scope and relevance of the review.

Addressing Publication Bias: Consider discussing how you plan to assess and address potential publication bias, such as using funnel plots or statistical tests. This would strengthen the credibility of the systematic review's findings.

Detailed Search Strategy: Although the manuscript outlines the databases to be searched, providing a detailed search strategy, including specific keywords and Boolean operators, could enhance transparency and replicability. This detail would also allow others to understand the comprehensiveness of your search.

Additional Sensitivity Analyses: Including additional sensitivity analyses, such as excluding studies with a high risk of bias or those with small sample sizes, could provide more nuanced insights into the robustness of your findings.

e.g

Cernat E M, Neagu A, Betianu C, Manolescu L, Avram G, Pogarasteanu M-E, Barbilian A. 2024. Balancing Anterior and Posterior Cruciate Ligaments in Adults. Cureus 16(5): e59683. DOI: 10.7759/cureus.59683

Cernat, E.M.; Dima, A.; Popescu, C.; Neagu, A.; Betianu, C.; Moga, M.; Manolescu, L.; Barbilian, A. Anterior Intercondylar Notch Geometry in Relation to the Native Anterior Cruciate Ligament Size. J. Clin. Med. 2024, 13, 309. https://doi.org/10.3390/jcm13020309

Consideration of Grey Literature: Including grey literature, such as conference abstracts, theses, and technical reports, could help mitigate publication bias and provide a more comprehensive view of the available evidence.

Patient and Public Involvement (PPI): If not already considered, the inclusion of patient and public involvement in the planning stages or interpretation of results could provide valuable insights and enhance the study's relevance to patient care.

Data Management Plan: While the manuscript states that all relevant data will be made available, providing a more detailed data management plan, including where and how the data will be stored and accessed, could clarify this aspect for potential users.

Future Research Directions: In the discussion section, it might be beneficial to outline potential areas for future research based on anticipated findings. This could help in identifying gaps that need to be addressed in subsequent studies.

These suggestions aim to enhance the study's transparency, reproducibility, and relevance to the field. The authors' consideration of these points could further strengthen the manuscript and its contributions to the literature on PCL injury treatments.

**********

what does this mean? ). If published, this will include your full peer review and any attached files.

If you choose “no”, your identity will remain anonymous but your review may still be made public.

**Do you want your identity to be public for this peer review?** For information about this choice, including consent withdrawal, please see our Privacy Policy

Reviewer #1: **Yes: ** Aissam Elmhiregh

Reviewer #2: No

**********

[NOTE: If reviewer comments were submitted as an attachment file, they will be attached to this email and accessible via the submission site. Please log into your account, locate the manuscript record, and check for the action link "View Attachments". If this link does not appear, there are no attachment files.]

While revising your submission, please upload your figure files to the Preflight Analysis and Conversion Engine (PACE) digital diagnostic tool, https://pacev2.apexcovantage.com/ . PACE helps ensure that figures meet PLOS requirements. To use PACE, you must first register as a user. Registration is free. Then, login and navigate to the UPLOAD tab, where you will find detailed instructions on how to use the tool. If you encounter any issues or have any questions when using PACE, please email PLOS at figures@plos.org

# Author comment: Author response to Decision Letter 1

**DOI:** 10.1371/journal.pone.0333015.r002

6 Aug 2025

We appreciate the corrections and suggestions made by the reviewers and the editor. We are sending the three requested files.

Thank you in advance.

Attachment Submitted filename: Response to Reviewers (1).pdf

# Peer review: Decision Letter 1

**DOI:** 10.1371/journal.pone.0333015.r003

**Authors:** Luciana Labanca

31 Aug 2025

Dear Dr. Sayum Filho,

plosone@plos.org . When you're ready to submit your revision, log on to https://www.editorialmanager.com/pone/ and select the 'Submissions Needing Revision' folder to locate your manuscript file.

- A rebuttal letter that responds to each point raised by the academic editor and reviewer(s). You should upload this letter as a separate file labeled 'Response to Reviewers'.
- A marked-up copy of your manuscript that highlights changes made to the original version. You should upload this as a separate file labeled 'Revised Manuscript with Track Changes'.
- An unmarked version of your revised paper without tracked changes. You should upload this as a separate file labeled 'Manuscript'.

If applicable, we recommend that you deposit your laboratory protocols in protocols.io to enhance the reproducibility of your results. Protocols.io assigns your protocol its own identifier (DOI) so that it can be cited independently in the future. For instructions see: https://journals.plos.org/plosone/s/submission-guidelines#loc-laboratory-protocols . Additionally, PLOS ONE offers an option for publishing peer-reviewed Lab Protocol articles, which describe protocols hosted on protocols.io. Read more information on sharing protocols at https://plos.org/protocols?utm_medium=editorial-email&utm_source=authorletters&utm_campaign=protocols .

We look forward to receiving your revised manuscript.

Kind regards,

Luciana Labanca

Academic Editor

PLOS ONE

Journal Requirements:

1. If the reviewer comments include a recommendation to cite specific previously published works, please review and evaluate these publications to determine whether they are relevant and should be cited. There is no requirement to cite these works unless the editor has indicated otherwise. 

2. Please review your reference list to ensure that it is complete and correct. If you have cited papers that have been retracted, please include the rationale for doing so in the manuscript text, or remove these references and replace them with relevant current references. Any changes to the reference list should be mentioned in the rebuttal letter that accompanies your revised manuscript. If you need to cite a retracted article, indicate the article’s retracted status in the References list and also include a citation and full reference for the retraction notice.

[Note: HTML markup is below. Please do not edit.]

Reviewers' comments:

Reviewer's Responses to Questions

**Comments to the Author**

1. Does the manuscript provide a valid rationale for the proposed study, with clearly identified and justified research questions?

Reviewer #3: Yes

Reviewer #4: Yes

**********

2. Is the protocol technically sound and planned in a manner that will lead to a meaningful outcome and allow testing the stated hypotheses?

Reviewer #3: Yes

Reviewer #4: Yes

**********

3. Is the methodology feasible and described in sufficient detail to allow the work to be replicable?

Reviewer #3: Yes

Reviewer #4: Yes

**********

4. Have the authors described where all data underlying the findings will be made available when the study is complete?

The PLOS Data policy

Reviewer #3: Yes

Reviewer #4: Yes

**********

5. Is the manuscript presented in an intelligible fashion and written in standard English?

Reviewer #3: Yes

Reviewer #4: Yes

**********

Please use the space provided to explain your answers to the questions above and, if applicable, provide comments about issues authors must address before this protocol can be accepted for publication. You may also include additional comments for the author, including concerns about research or publication ethics.

You may also provide optional suggestions and comments to authors that they might find helpful in planning their study.

Reviewer #3: I would like to congratulate the authors for the thorough revisions performed in response to the previous round of comments. All the requested modifications were adequately addressed, and the manuscript is now much clearer and very well written. The study is of high relevance, and I believe it will be of interest to the readership of PLOS ONE.

Only a few minor points remain to be clarified before acceptance:

Introduction – Introduction – Although well contextualized, the introduction is somewhat extensive. Consider condensing it and improving the flow by creating stronger connections between paragraphs, as they are currently too short.

Intervention and control – The distinction between what is considered intervention and control remains slightly unclear. Please provide a more explicit description in the Methods section.

Study management software – Kindly specify whether any software was used to manage the study selection process, particularly for duplicate removal and screening. This will strengthen the transparency and reproducibility of the review.

Reviewer #4: This protocol has a clear structure and detailed content, but there is still room for improvement, as specified below:

1. The depth of background information is insufficient. Although it mentions that "conservative treatment and surgical treatment each have advantages and disadvantages", it fails to briefly explain the existing research consensus on the "differences in applicable populations" and "preliminary trends in short-term and long-term efficacy" between the two types of treatment. This makes it difficult to highlight the necessity of the study. It is recommended to supplement the content to further emphasize the value of conducting this systematic review.

2. It is suggested to revise "a systematic review protocol" to "a systematic review and meta-analysis protocol", which can more accurately reflect the study design.

3. For "over 17 years of age" → it is recommended to clearly state "≥18 years" to avoid ambiguity.

4. In the Outcomes section, since Pain is listed as a primary outcome, it is advisable to specify the measurement tool (e.g., Visual Analog Scale (VAS) score).

5. In the Search Strategy section, the search period ends in April 2024, while the study is scheduled to start in August 2025. Please provide an explanation for this discrepancy. It is recommended to extend the search end date to the present.

6. Please check the entire text for spelling and grammatical errors. For example, "Strengths and Limitations os this Study" should be corrected to "Strengths and Limitations of this Study".

**********

what does this mean? ). If published, this will include your full peer review and any attached files.

If you choose “no”, your identity will remain anonymous but your review may still be made public.

**Do you want your identity to be public for this peer review?** For information about this choice, including consent withdrawal, please see our Privacy Policy

Reviewer #3: No

Reviewer #4: No

**********

[NOTE: If reviewer comments were submitted as an attachment file, they will be attached to this email and accessible via the submission site. Please log into your account, locate the manuscript record, and check for the action link "View Attachments". If this link does not appear, there are no attachment files.]

While revising your submission, please upload your figure files to the Preflight Analysis and Conversion Engine (PACE) digital diagnostic tool, https://pacev2.apexcovantage.com/ . PACE helps ensure that figures meet PLOS requirements. To use PACE, you must first register as a user. Registration is free. Then, login and navigate to the UPLOAD tab, where you will find detailed instructions on how to use the tool. If you encounter any issues or have any questions when using PACE, please email PLOS at figures@plos.org

# Author comment: Author response to Decision Letter 2

**DOI:** 10.1371/journal.pone.0333015.r004

7 Sep 2025

Thanks for the suggestions and corrections. We tried to respond and correct all of them. We are sending 3 files: "response to reviewers", "Revised manuscript with track changes" and "Manuscript".

Attachment Submitted filename: 2 Response to Reviewers.docx

# Editor report: Decision Letter 2

**DOI:** 10.1371/journal.pone.0333015.r005

**Authors:** Luciana Labanca

9 Sep 2025

Interventions for treating posterior cruciate ligament injuries of the knee in adults: a systematic review and meta-analysis protocol.

PONE-D-24-10653R2

Dear Dr. Sayum Filho,

We’re pleased to inform you that your manuscript has been judged scientifically suitable for publication and will be formally accepted for publication once it meets all outstanding technical requirements.

Within one week, you’ll receive an e-mail detailing the required amendments. When these have been addressed, you’ll receive a formal acceptance letter and your manuscript will be scheduled for publication.

An invoice will be generated when your article is formally accepted. Please note, if your institution has a publishing partnership with PLOS and your article meets the relevant criteria, all or part of your publication costs will be covered. Please make sure your user information is up-to-date by logging into Editorial Manager at Editorial Manager®  and clicking the ‘Update My Information' link at the top of the page. For questions related to billing, please contact billing support .

If your institution or institutions have a press office, please notify them about your upcoming paper to help maximize its impact. If they’ll be preparing press materials, please inform our press team as soon as possible -- no later than 48 hours after receiving the formal acceptance. Your manuscript will remain under strict press embargo until 2 pm Eastern Time on the date of publication. For more information, please contact onepress@plos.org.

Kind regards,

Luciana Labanca

Academic Editor

PLOS ONE

Additional Editor Comments (optional):

Reviewers' comments:

# Editor report: Acceptance letter

**DOI:** 10.1371/journal.pone.0333015.r006

**Authors:** Luciana Labanca

PONE-D-24-10653R2

PLOS ONE

Dear Dr. Sayum Filho,

I'm pleased to inform you that your manuscript has been deemed suitable for publication in PLOS ONE. Congratulations! Your manuscript is now being handed over to our production team.

At this stage, our production department will prepare your paper for publication. This includes ensuring the following:

* All references, tables, and figures are properly cited

* All relevant supporting information is included in the manuscript submission,

* There are no issues that prevent the paper from being properly typeset

You will receive further instructions from the production team, including instructions on how to review your proof when it is ready. Please keep in mind that we are working through a large volume of accepted articles, so please give us a few days to review your paper and let you know the next and final steps.

Lastly, if your institution or institutions have a press office, please let them know about your upcoming paper now to help maximize its impact. If they'll be preparing press materials, please inform our press team within the next 48 hours. Your manuscript will remain under strict press embargo until 2 pm Eastern Time on the date of publication. For more information, please contact onepress@plos.org.

You will receive an invoice from PLOS for your publication fee after your manuscript has reached the completed accept phase. If you receive an email requesting payment before acceptance or for any other service, this may be a phishing scheme. Learn how to identify phishing emails and protect your accounts at https://explore.plos.org/phishing.

If we can help with anything else, please email us at customercare@plos.org.

Thank you for submitting your work to PLOS ONE and supporting open access.

Kind regards,

PLOS ONE Editorial Office Staff

on behalf of

Dr. Luciana Labanca

Academic Editor

PLOS ONE
